# Sensorineural correlates of failed functional recovery after natural regeneration of vestibular hair cells in adult mice

**DOI:** 10.3389/fneur.2024.1322647

**Published:** 2024-03-08

**Authors:** Emmanuel J. Jáuregui, Kelli L. Scheinman, Ingrid K. Bibriesca Mejia, Lindsay Pruett, Hannah Zaini, Connor Finkbeiner, Jonathan A. Phillips, Jay A. Gantz, Tot Bui Nguyen, James O. Phillips, Jennifer S. Stone

**Affiliations:** Department of Otolaryngology-Head and Neck Surgery and the Virginia Merrill Bloedel Hearing Research Center, University of Washington, Seattle, WA, United States

**Keywords:** vestibular, hair cell, VOR, mice, regeneration, cFos

## Abstract

Vestibular hair cells (HCs) are mechanoreceptors that sense head motions by modulating the firing rate of vestibular ganglion neurons (VGNs), whose central processes project to vestibular nucleus neurons (VNNs) and cerebellar neurons. We explored vestibular function after HC destruction in adult *Pou4f3^+/DTR^ (DTR)* mice, in which injections of high-dose (50 ng/g) diphtheria toxin (DT) destroyed most vestibular HCs within 2 weeks. At that time, *DTR* mice had lost the horizontal vestibulo-ocular reflex (aVOR_H_), and their VNNs failed to upregulate nuclear cFos expression in response to a vestibular stimulus (centrifugation). Five months later, 21 and 14% of HCs were regenerated in utricles and horizontal ampullae, respectively. The vast majority of HCs present were type II. This degree of HC regeneration did not restore the aVOR_H_ or centrifugation-evoked cFos expression in VNNs. The failure to regain vestibular pathway function was not due to degeneration of VGNs or VNNs because normal neuron numbers were maintained after HC destruction. Furthermore, sinusoidal galvanic stimulation at the mastoid process evoked cFos protein expression in VNNs, indicating that VGNs were able to regulate VNN activity after HC loss. aVOR_H_ and cFos responses in VNNs were robust after low-dose (25 ng/g) DT, which compared to high-dose DT resulted in a similar degree of type II HC death and regeneration but spared more type I HCs in both organs. These findings demonstrate that having more type I HCs is correlated with stronger responses to vestibular stimulation and suggest that regenerating type I HCs may improve vestibular function after HC loss.

## 1 Introduction

The vestibular system relies on mechanosensory receptors called hair cells (HCs) that sense head motions. In mammals, HCs in utricles and saccules detect linear accelerations (head tilt and translation), and HCs in horizontal, anterior, and posterior ampullae respond to angular accelerations (head rotations). The HC’s sensory organelle is a bundle of stiff hair-like structures whose displacement during head acceleration alters the HC membrane potential. This leads to changes in the firing rate of vestibular ganglion neurons (VGNs), upon which the HCs synapse. Action potentials are conveyed along VGN axons (the vestibular nerve), which terminate on vestibular nucleus neurons (VNNs) in the brainstem or on cerebellar neurons (primarily in the flocculonodular lobe and caudal vermis). From these regions, signals originating in the inner ear are relayed to other brain regions that control body movements, spatial orientation, and additional functions critical to our sense of wellbeing.

In humans, vestibular HCs degenerate during aging (1, 2) and after exposure to ototoxins such as aminoglycoside antibiotics (3). Significant loss of vestibular HCs leads to debilitating symptoms such as vertigo, restricted mobility, destabilized vision, and imbalance. Balance dysfunction is common, affecting about a third of people over the age of 40 (4). The risk of falling is increased in people with vestibular dysfunction, and falls are a leading cause of death in older people (5, 6). Humans may regenerate small numbers of vestibular HCs (7), but this process does not seem to restore vestibular function. Guinea pigs and mice regenerate substantial numbers of HCs in utricles, saccules, and ampullae after ototoxin- or genetically-mediated HC damage (8–13). For example, nearly 20% of utricular HCs are replaced after near-complete HC destruction in adult mice (11). Despite this natural repair, rodents still exhibit vestibulo-motor deficits (data not shown) for months after HCs have been replaced (11). We do not yet understand why natural HC regeneration fails to restore vestibulo-motor functions, but it is notable that only one type of vestibular HC – type II – is naturally replaced after damage [for example (8, 10, 11)].

In this study, we examined some aspects of vestibular function after natural HC regeneration in adult mice. We measured the angular vestibulo-ocular reflex in the horizontal plane (aVOR_H_). Such head rotations stimulate HCs in the horizontal ampulla, which via a subset of VNNs drive oculomotor and abducens motor neurons to move the eyes in the horizontal plane in directions opposite to head motions, stabilizing vision. We also measured cFos expression in medial and spinal VNNs in freely moving mice subjected to centrifugation around the earth-vertical axis, which likely altered HC activity in several or all vestibular organs. cFos is an immediate early transcriptional activator whose expression may increase during changes in cellular activity (14–16). cFos has been used to study VNN responses to a variety of vestibular stimuli including linear and rotational accelerations that activate otolithic and/or semicircular canal afferents in rodents and other species [for example (17–22)].

We found that neither the aVOR_H_ nor centrifugation-evoked cFos in VNNs was restored when sensory organs were populated by regenerated type II HCs and only rare surviving type I HCs. However, these responses were present when approximately half of the type I HC population survived DT treatment. These findings suggest that stimulating regeneration of type I HCs in adult mammals may significantly improve the restoration of vestibular function.

## 2 Materials and methods

### 2.1 Mice

To destroy HCs, we used *Pou4f3^DTR^* mice, in which the human *diphtheria toxin receptor (DTR)* gene is inserted into exon 1 of *Pou4f3* (11, 23). Experimental mice were heterozygotes (*Pou4f3*^+^*/^DTR^*, *c*alled *DTR*) with a C57BL/6J background, injected with diphtheria toxin (DT). Controls consisted of wildtype (*Pou4f3*^+^*/*^+^, abbreviated to WT) littermates that did or did not receive DT, or *DTR* mice that were injected with saline. Male and female mice were used between 6 weeks and 1.5 years of age.

Mice were housed with access to food and water. Experiments were approved by the Institutional Animal Care and Use Committee of the University of Washington School of Medicine (Seattle, WA) and adhered to standards of the American Veterinary Medical Association.

### 2.2 DT or saline administration

Adult mice (6–16 weeks of age) received two intramuscular (IM) injections of DT (List Biological Laboratories, Campbell, CA) at either 25 ng/g (low-dose) or 50 ng/g (high-dose), spaced 2 days apart. Some control mice were administered IM saline.

### 2.3 VOR measurements

We measured the angular vestibulo-ocular reflex in the earth-horizontal plane (aVOR_H_) in control and experimental mice. We examined 4 groups of controls between the ages of 2 and 9 months: (1) WT mice with no injection (*n* = 3); (2) *DTR* mice given saline (*n* = 3); (3) WT mice given low-dose DT (*n* = 6); and (4) WT mice given high-dose DT (*n* = 4). We examined 6 groups of experimental mice: *DTR* mice given low-dose DT, which induced moderate hair cell loss, at (1) 14d (*n* = 3), (2) 70d (*n* = 2), or (3) 140–170d post-DT (*n* = 6); and *DTR* mice given high-dose DT, which induced severe hair cell loss, at (1) 14d (*n* = 3), (2) 70d (*n* = 5), or (3) 140–170d post-DT (*n* = 8).

We used a customized, silent linear servo-driven VOR testing apparatus that enables *en bloc* rotation of adult mice about an earth-vertical axis that intersects the interaural axis in the mid sagittal plane. The mouse was oriented in the stereotaxic plane, and the eye was imaged with IR video recording. Methods generally followed those described in Stahl et al. (24) or Faulstich et al. (25). Each mouse was implanted with a head-post to orient the head and to limit head movements using an apparatus similar to that described in Kaneko et al. (26). Mice were deeply anesthetized with isoflurane. A small incision was made in the scalp, and an acrylic head-post was affixed to the skull using light-cured dental composite (Ortho-Jet, Lang Dental). VOR testing was performed 1–4 days later. Mice were anesthetized with isoflurane and placed in a plastic restraining tube that encircled their body. Mice were administered 1% pilocarpine to constrict pupils and enable eye-tracking. After recovery from anesthesia for approximately 5 min, mice were set in the VOR testing apparatus. The head-post was affixed to a rate table inside a light-tight enclosure containing a high magnification IR CCD camera, and VOR testing was initiated. We used video-oculography to measure eye position (aVOR_H_) during sinusoidal rotation about an earth vertical axis with an amplitude of +/− 10° in complete darkness. Stimulus frequency ranged from 0.3 to 1.0 Hz. Eye movements during epochs of stable head position (relative to the rate table) were analyzed to determine the gain of the aVOR_H_. Slow-phase eye velocity was calculated offline from differentiation of filtered eye position traces that were de-saccaded based on a velocity criterion using custom software. aVOR_H_ gains (eye velocity/head velocity) were calculated using a least-squares fit of accumulated eye and head velocity data to a sine wave, for multiple cycles of rotation. Confirmation of results was obtained by direct measurement of the amplitude of sinusoidal eye movements elicited during cycles without saccadic eye movement, compared to the constant amplitude of head rotation.

After VOR measurements were completed, mice were euthanized, and inner ear organs were prepared for immunolabeling.

### 2.4 Centrifugal stimulation of vestibular hair cells

Each mouse was placed in a 500 mL beaker at the periphery of a rate table with a 28 cm radius and subjected to sinusoidal centrifugation about an earth-vertical axis at 0.01 Hz, pk-pk 600°/s, for 10 min. During centrifugation, mice could move freely in the beaker. With this stimulus, mice likely experienced acceleration and deceleration in multiple planes, which we expected to alter HC activity in several vestibular organs and therefore to modulate both rotational and linear acceleration afferents. Because mice were not restrained, this stimulus was stochastic and variable across mice.

Following the stimulation period, mice recovered in a cage with food and water for 45 min. Then, they were deeply anesthetized with ketamine (100 mg/kg) and xylazine (5 mg/kg) delivered intraperitoneally (IP), and cardiac perfusion was performed with normal saline for a few seconds followed by 4% paraformaldehyde (PFA, Sigma-Aldrich) fixative for a few minutes. In pilot studies, we noted no qualitative difference in numbers of cFos-labeled neurons when we waited 45, 60, or 90 min after centrifugation to initiate cardiac perfusion.

We tested four groups of control mice, all between 2 and 16 months of age, and *n* = 3 for all groups: (1) WT mice with no DT and no centrifugation; (2) *DTR* mice with no DT and no centrifugation; (3) WT mice with no DT but with centrifugation); and (4) WT mice centrifuged 78d after high-dose DT. We tested four groups of experimental mice: *DTR* mice with high-dose DT and tested at (1) 14d post-DT, (2) 78d post-DT, or (3) 360d (1 year) post-DT; and (4) *DTR* mice with low-dose DT and tested at 1-year post-DT.

### 2.5 Sinusoidal galvanic vestibular stimulation

Mice were subjected to sinusoidal galvanic stimulation (sGVS) at the mastoid process, similar to previously published methods (27, 28). Mice were deeply anesthetized with ketamine and xylazine as described above. Current was delivered via Ag/AgCl needle electrodes (F-E2-48 Genuine Grass Reusable Platinum Subdermal) inserted into the post-auricular skin overlying the mastoid process on both sides of the mouse. sGVS (cathodal current alternating from one side of the head to the other) was applied for 5 sinusoidal cycles, each 40 s in duration (0.025 Hz), and was repeated 5 times with a 3-min rest between each repetition. Each stimulation session lasted 29 min per mouse. sGVS currents were generated by a linear stimulus isolator (WPI A395) at 2 mA peak current modulated by a wave form function generator (Siglent Technologies, SDG 1032X). Sixty minutes after sGVS, mice were deeply anesthetized with ketamine and xylazine and subjected to cardiac perfusion of PFA (described above).

We performed sGVS on several groups of mice between 2 and 9 months of age: WT mice with no DT (*n* = 4); WT mice (*n* = 3) and *DTR* mice (*n* = 3) at 14d pDT; WT mice (*n* = 5) and *DTR* mice (*n* = 4) at 78d pDT; and WT mice (*n* = 3) and *DTR* mice (*n* = 5) at 180d pDT. For this experiment, all DT doses were high (50 ng/g).

### 2.6 Brain sectioning and labeling

After cardiac perfusion with PFA, mouse brains were immersion-fixed overnight at 4 ^°^C then switched to phosphate buffered saline (PBS) at 4^°^C. Whole brains were mounted in agar gel, and 50 μm serial sections spanning the entire vestibular nucleus (VN) complex were cut in the coronal (transverse) plane using a vibratome (Leica). We collected brainstem sections, starting at the caudal edge of the fourth ventricle (Bregma −7.08 mm) and ending around Bregma −5.68 mm (29). All sections were stored in PBS at 4°C.

Every third section was immunohistochemically labeled for phosphorylated cFos using standard immunohistochemistry at room temperature. Sections were treated with 30% H_2_O_2_ diluted in PBS for 30 min to quench endogenous peroxidases. Sections were incubated in blocking solution consisting of 10% normal horse serum diluted in PBT (PBS plus 0.1% TritonX100) for 1–3 h. Sections were treated with primary antibody (rabbit anti-phospho-cFos IgG; RRID:AB_2106617; Cell Signaling Technology #4384) diluted 1:500 in blocking solution for 12–36 h. After rinsing with PBS, sections were incubated in secondary antibody (biotinylated goat anti-rabbit IgG; Vector Laboratories #BA-100) diluted 1:300 in blocking solution for 1–2 h. ABC reagent (Vector Laboratories #PK-4000) was applied for 45 min −1 h. Sections were immersed in 3–3′ diaminobenzidine (0.05%) and 1% H_2_O_2_ dissolved in 0.05 M Tris–HCL for 1–5 min. Sections adjacent to cFos-labeled sections were stained for Nissl substance using 0.1–1.0% thionin acetate (Sigma Aldrich) to (1) identify anatomical boundaries of each VNN and (2) measure VNN density (see below). Sections were mounted on Superfrost Plus slides (Fisher Scientific, #22-037-246) with DPX media (Electron Microscopy Sciences, #13512) and coverslipped.

In some sections, cFos, ßIII tubulin (Tubb3), and/or Pou4f3 were labeled using immunofluorescence. Methods for labeling cFos were the same as above except no H_2_O_2_ was added, and cFos primary antibody was followed by Alexa Fluor-conjugated secondary antibody (Invitrogen). Sections were co-labeled with rabbit anti-ßIII tubulin (Tubb3; 1:500, RRID:AB_291637; #PRB-435P) and detected with 1:300 Alexa Fluor-conjugated secondary antibody (Invitrogen). To detect Pou4f3, sections were labeled with rabbit anti-Pou4f3 (1,300, RRID, AB_2878872; #21509-1-AP; Proteintech) and detected with Alexa Fluor-conjugated secondary antibody.

### 2.7 Microscopic analysis and quantification of cFos-labeled neurons in vestibular nucleus neurons (VNNs)

Imaging of VNNs labeled for cFos following either centrifugation or sGVS was performed using a Zeiss Axioplan or Nikon Axiophot microscope with 4x-20x objectives in brightfield mode. Image capture software was Slidebook (Intelligent Imaging Innovations, Denver, CO). Counts of cFos-labeled VNNs were performed at 20x using an eye piece reticle with a 10 × 10 grid. Nissl-stained sections and a stereotaxic mouse brain atlas (29) were consulted when assigning cells to one VNN or another. Every stained section along the rostro-caudal axis of the VN complex was analyzed. We counted VNNs with strong nuclear cFos labeling in every section that contained the medial vestibular (MeV) or spinal vestibular (SpV) nucleus. cFos-labeled neurons in left and right sides of the brain were counted; counts for each side were averaged.

Imaging of VNNs labeled for cFos and Tubb3 was performed using an Olympus FV-1000 confocal microscope with a 60x oil objective.

### 2.8 Brain mapping of cFos-labeled VNNs

Positions of cFos-labeled cells in MeV and SpV nuclei were recorded onto template maps of the brainstem created by scanning and digitizing images from a mouse brain stereotaxic atlas (29).

### 2.9 Quantification of vestibular ganglion neurons (VGNs)

Following cardiac perfusion with PFA, we isolated the temporal bone, maintaining a bit of lateral brainstem and cerebellum to preserve the central processes of the eight nerve and the vestibular ganglion neurons. We decalcified bones using EDTA and post-fixed them with 2% osmium tetroxide in sodium cacodylate buffer for 1 h. We rinsed and stored tissue in the same buffer at 4°C until embedding. At that point, tissue was dehydrated in graded ethanol and propylene oxide, then embedded in Eponate (Ted Pella Inc. #18010). Blocks were trimmed, oriented, and sectioned in the near-coronal plane at 2 μm. Sections were labeled with toluidine blue (Sigma-Aldrich #T3260), dried, and coverslipped using DPX.

Using a Zeiss Axioplan microscope, we took digital images with a 20x objective of every fifth section (sampling every 10 μm) through the entire vestibular ganglion including superior and inferior portions. Cells were considered neurons if they had a large nucleus, a single prominent nucleolus, and cytoplasmic Nissl substance. In each section, we counted VGNs using the CellCounter plugin in Fiji, only including cells with a visible nucleolus. We measured the VGN area as defined by the area occupied by neuronal cell bodies. Cell density was expressed as numbers of neurons per 10,000 μm^2^. We analyzed WT mice with no DT (*n* = 2 mice), WT mice at 170d pDT at 50 ng/g (*n* = 3 mice), and *DTR* mice at 170d post-DT at 50 ng/g (*n* = 3 mice).

### 2.10 Quantification of vestibular nucleus neurons (VNNs)

We counted VNNs in brainstem slices generated using a vibratome and Nissl-stained. We analyzed 3 WT mice and 3 *DTR* mice, all at 400d post-DT. We counted Nissl-positive neurons per field using a Zeiss Axioplan microscope under brightfield microscopy with a 20x objective and a 10 × 10 eye-piece reticule. Classification as neurons resembled those for VGNs but some VNNs had well defined dendrites, helping to confirm their neural identity. In each section, we counted VNNs using the same methods as for VGNs. To standardize sampling, counts were performed at three specific anatomical locations: at the caudal start of 4th ventricle (Bregma −7.08 mm), at the caudal start of dorsal cochlear nucleus (Bregma −6.48 mm), and at the caudal start of inferior cerebellar peduncle (Bregma −5.88 mm).

### 2.11 Immunolabeling of inner ear organs

Mice were deeply anesthetized using inhaled CO_2_ or intraperitoneal sodium pentobarbital solution and killed by decapitation. Temporal bones were rapidly dissected from head, and 4% PFA was perfused into the vestibule to rapidly fix the organs. Utricles and horizontal ampullae were isolated, and the capsule, otoconia, otoconial membrane, and cupula were dissected away. After storage in PBS at 4°C, organs were incubated for 30 min in blocking solution (2% bovine serum albumin, 0.8–5% normal horse serum in PBT). Organs were incubated overnight at room temperature in primary antibody diluted 1/1500 in blocking solution: (1) rabbit anti-myosin VIIa (RRID:AB_10013626; #25–6790; Proteus Biosciences Inc.) and (2) chicken anti-neurofilament 200 KD (RRID:AB_11212161, Millipore, #5539) to label the afferent calyx found only on type I HCs. After rinsing with PBS, organs were treated with secondary antibodies conjugated to fluorescent molecules (Invitrogen, Sigma-Aldrich) diluted 1/300 in blocking solution for 2–3 h. To label cell nuclei, organs were soaked in 4′,6-diamidino- 2-phenylindole (DAPI, Sigma-Aldrich #28718–90-3) at 1 μg/ml diluted in PBS for 10 min, mounted onto slides with FluoroMount-G (#F4680, Sigma-Aldrich), and coverslipped.

### 2.12 Quantification of vestibular hair cells in utricles and horizontal ampullae

Fluorescent imaging of the sensory epithelia of utricles and horizontal ampullae was performed using an Olympus FV-1000 confocal microscope. Z-series images from the epithelial surface through the stromal connective tissue were obtained using a 20x objective. In addition, a 60x oil objective was used to image the entire sensory epithelium for HC counts. Using Fiji’s CellCounter plugin, we counted all myosin VIIa-positive cells per field (sampling from approximately 25% of the area of each organ). For counts of type I versus type II HCs, we used standard morphological criteria (30, 31). In brief, type I HCs have a thin neck, small round nucleus, and round cell body with relatively little cytoplasmic myosin VIIa staining. They have tall stereocilia, and their cell bodies are surrounded by a neurofilament-labeled calyx afferent terminal. By contrast, type II HCs have thick necks and large basolateral cytoplasmic processes with strong myosin VIIa labeling.

### 2.13 Graphs and statistical analysis

Graphs were generated using Prism 6.0 (GraphPad). Data are expressed as mean ± standard error of the mean (SEM). Numerical data were analyzed using one-way ANOVA or two-way ANOVA followed by Tukey’s multiple comparisons test, or using Student’s *t*-test, as described in Results. Differences were considered significant if *p* ≤ 0.05. Animal numbers are provided in the description for each quantitative analysis (above) and in the graphs and tables. For hair cell counts, we collected data from one utricle and one horizontal ampulla per mouse. Both the left and right organs from *DTR* mice exhibited similar degrees of HC loss and regeneration after DT treatment (not shown).

Data collection and analysis for VOR and cFos measurements were blinded, as were neuron counts. However, it was not possible to blind experimenters to HC counts because it was readily evident upon image inspection which samples had no HC loss, moderate HC loss, or near-complete HC loss.

## 3 Results

### 3.1 High-dose (50 ng/g) DT destroyed most vestibular HCs in adult DTR mice, and only type II HCs were regenerated

We characterized the HC lesion in utricles and horizontal ampullae before testing vestibular function. To kill HCs, we used *Pou4f3^+/DTR^* (abbreviated to *DTR*) mice, in which the human *DTR* coding sequence is inserted into exon 1 of *Pou4f3* (11), which is expressed in all HCs (32, 33). Because *Pou4f3* expression is limited to HCs, it is believed that other cells in the vestibular pathway are spared.

For initial experiments, *DTR* mice received two injections of high-dose DT (50 ng/g), spaced 2 days apart. We analyzed five sets of control mice that were expected to have normal HC numbers (11): *Pou4f3^+/+^* (WT) mice with no DT treatment, WT mice at 14 days (d), 70d, and 140d after high-dose DT, and *DTR* mice with saline. Whole-mount organs were labeled to detect myosin VIIa, a selective marker of HCs, and all HCs were counted.

**Figure 1:**
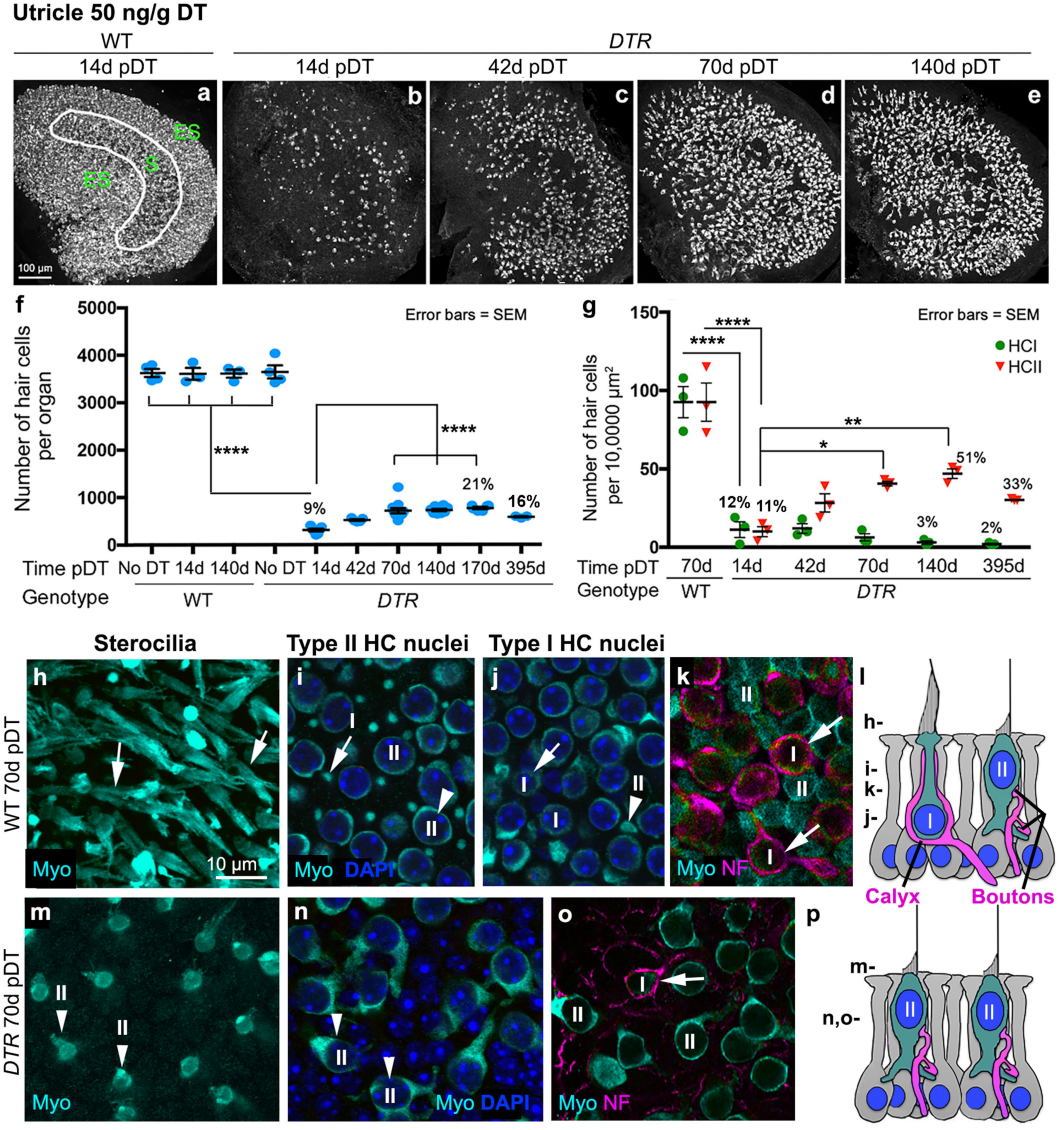
Most HCs in utricles were killed by 50 ng/g DT, and about half of the type II HC population was regenerated. **(A–E)** Images in the xy plane (parallel to lumen) of utricles labeled for hair cell (HC) marker myosin VIIa (Myo, white) from wildtype (WT) mice at 14d post (p)-diphtheria toxin (DT) **(A)** and from *Pou4f3^DTR^(DTR)* mice at 14d **(B)**, 42d **(C)**, 70d **(D)**, and 140d **(E)** post-DT at 50 ng/g. The epithelial zones are indicated in panel A: ES, extrastriolar. S, striolar. **(F)** Graph shows mean number (± standard error of the mean, or SEM) of Myo-labeled HCs (type I and II) from four sets of control (undamaged) mice (WT with no DT, WT at 14d post-DT, WT at 140d post-DT, and *DTR* mice with no DT) and from experimental (*DTR*, damaged) mice from 14d to 395d pDT. **(G)** Graph shows the mean number (±SEM) of type I (green) and type II (red) HCs per 10,000 μm^2^ in WT mice at 70d post-DT and in *DTR* mice at times ranging from 14d to 395d post-DT. For both graphs, each symbol is the mean from a single mouse, and % values represent % of control (also see Tables 1, 2). One-factor ANOVA revealed significant differences between some groups, with lines describing each comparison and asterisks showing significance level for each comparison (*p ≤ 0.05, **p ≤ 0.01, ****p ≤ 0.0001). To simplify the graphs, only the most pertinent significant differences are shown; additional significant differences are presented in Tables 1, 2. **(H–K)** Confocal slices from one field in the lateral extrastriolar region from a WT mouse at 70d post-DT, taken in the xy plane at levels shown in panel l. Utricles were labeled for myosin VIIa (Myo, cyan) and DAPI (blue). Arrows show type I HCs, and arrowheads points type II HCs at each level. Panels **(I,J)** are from different focal points in the same field. Panel **(K)** shows neurofilament (NF)-labeled (magenta) afferent calyces (arrows) surrounding each type I HC (Myo, cyan) but not the type II HC. **(L)** Schematic representation of a cross section of the normal sensory epithelium showing type I and type II HCs (cyan cytoplasm, blue nucleus), afferent terminals (magenta), and supporting cells (light gray cytoplasm, blue nucleus). The level of each slice shown in panels H-K is indicated on the left. **(M,N)** Confocal slices from one field in the extrastriola from a *DTR* mouse at 70d post-DT (50 ng/g) labeled for Myo (cyan) and DAPI (blue) shown at the levels indicated in panel **(L)**. Arrowheads in panels M,N point to type II HCs, at different focal points in the same field. **(O)** NF-labeled (magenta) calyx (arrow) surrounding a surviving type I HC (Myo, cyan) but not type II HCs. **(P)** Schematic representation of a cross section of a regenerated utricular sensory epithelium showing type I and type II HCs (cyan cytoplasm, blue nucleus), afferent terminals (magenta), and supporting cells (light gray cytoplasm, blue nucleus). The level of each slice shown in panels M-O is indicated on the left. I or HCI = type I hair cell; II or HCII = type II hair cell.

**Figure 2:**
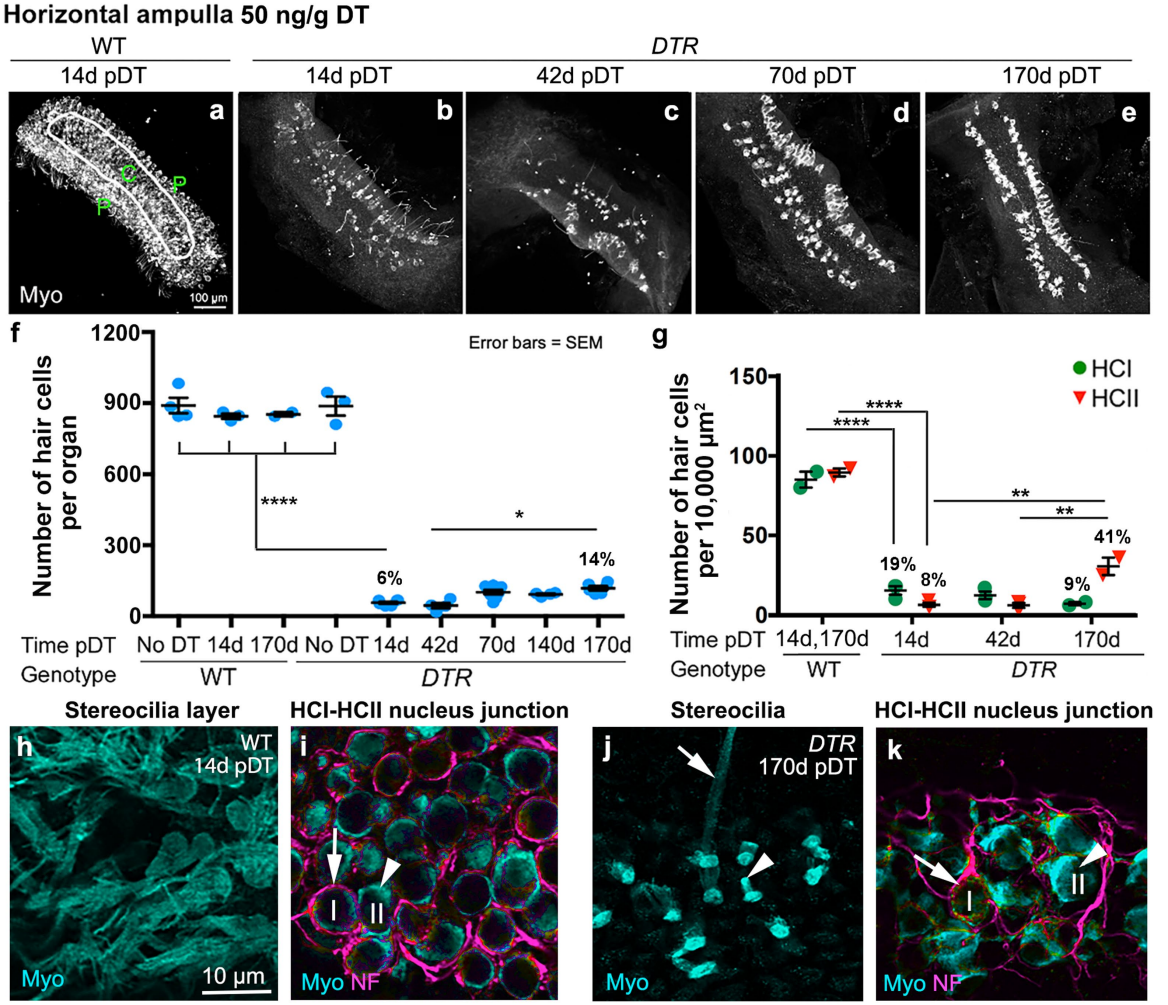
Most HCs in horizontal ampullae were killed by 50 ng/g DT, and about half of the type II HC population was regenerated. **(A–E)** Images in the xy plane (parallel to lumen) of utricles labeled for hair cell (HC) marker myosin VIIa (Myo, white) from wildtype (WT) mice at 14d post (p)-diphtheria toxin (DT) **(A)** and from *Pou4f3^DTR^(DTR)* mice at 14d **(B)**, 42d **(C)**, 70d **(D)**, and 170d **(E)** post-DT at 50 ng/g. The epithelial zones are indicated in panel **(A)**: C, central. P, peripheral. **(F)** Graph shows mean number (± standard error of the mean, or SEM) of Myo-labeled HCs (type I and II) from four sets of control (undamaged) mice (WT with no DT, WT at 14d post-DT, WT at 170d post-DT, and *DTR* mice with no DT) and from experimental (*DTR*, damaged) mice from 14d to 170d pDT. **(G)** Graph shows the mean number (±SEM) of type I (green) and type II (red) HCs per 10,000 μm^2^ in WT mice at 14d or 170d post-DT and in *DTR* mice at times ranging from 14d to 170d post-DT. For both graphs, each symbol is the mean from a single mouse. % values represent % of control (also see Tables 1, 2). One-factor ANOVA revealed significant differences between some groups, with lines describing each comparison and asterisks showing significance level for each comparison (*p ≤ 0.05, **p ≤ 0.01, ****p ≤ 0.0001). To simplify the graphs, only the most pertinent significant differences are shown; additional significant differences are presented in Tables 1, 2. **(H,I)** Confocal slices from one field in the peripheral region from a WT mouse at 14d post-DT, taken in the xy plane. Ampulla was labeled for myosin VIIa (Myo, cyan), neurofilament (NF, magenta) and DAPI (blue). Arrow in panel i points to type I HC (with a calyx) and arrowhead points to type II HC (no calyx). **(J,K)** Confocal slices from one field in the peripheral region from a *DTR* mouse at 170d post-DT. Arrows in panels **(J,K)** (taken from different focal planes in the same field) point to a type I HC (long stereeocilia, with a calyx), and arrowheads point to a type II HC (short stereocilia, no calyx), at each level. I or HCI = type I hair cell; II or HCII = type II hair cell.

**Figure 3:**
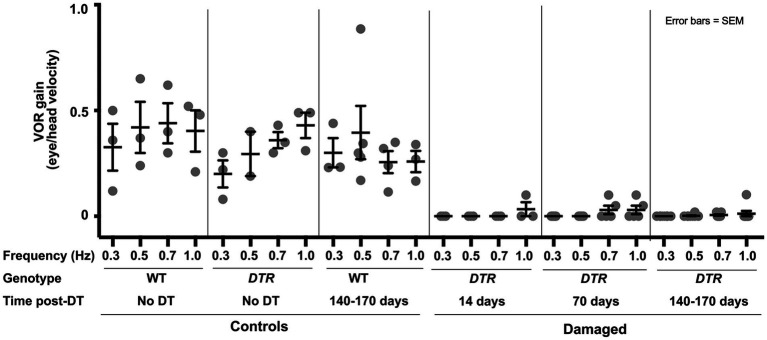
The horizontal angular vestibulo-ocular reflex was lost after HC destruction by 50 ng/g DT and was not restored after type II HC regeneration. Graph shows mean (±SEM) gains of the aVOR_H_ following head rotations in the dark at a range of frequencies (0.3 to 1.0 Hertz, or Hz) for three sets of control mice wildtype (WT) mice with no DT, *DTR* mice with no DT, and WT mice at 140-170d post-DT and for three sets of experimental (damaged) mice (*DTR* mice at 14d, 70d, and 140-170d post-DT). Each dot is the mean from a single mouse. Analysis of gains at each frequency with one-factor ANOVA revealed the following. There were no significant differences amongst the three controls at any frequency except, at 0.7 Hz, the WT no DT control was significantly different from the DTR no DT group (*p* ≤ 0.05). There were no significant differences amongst the three experimental groups at any frequency. All control groups were significantly different from all damaged groups at the same frequency (*p* values ranging from ≤0.05 to ≤0.0001, with the following exceptions: at 0.5 Hz, there was no significant difference between DTR no DT controls and any experimental group [*DTR* mice] at 14, 70, or 140-170d post-DT), and at 0.3 Hz, there was no significant difference between DTR no DT controls and *DTR* mice at 14d post-DT.

**Figure 4:**
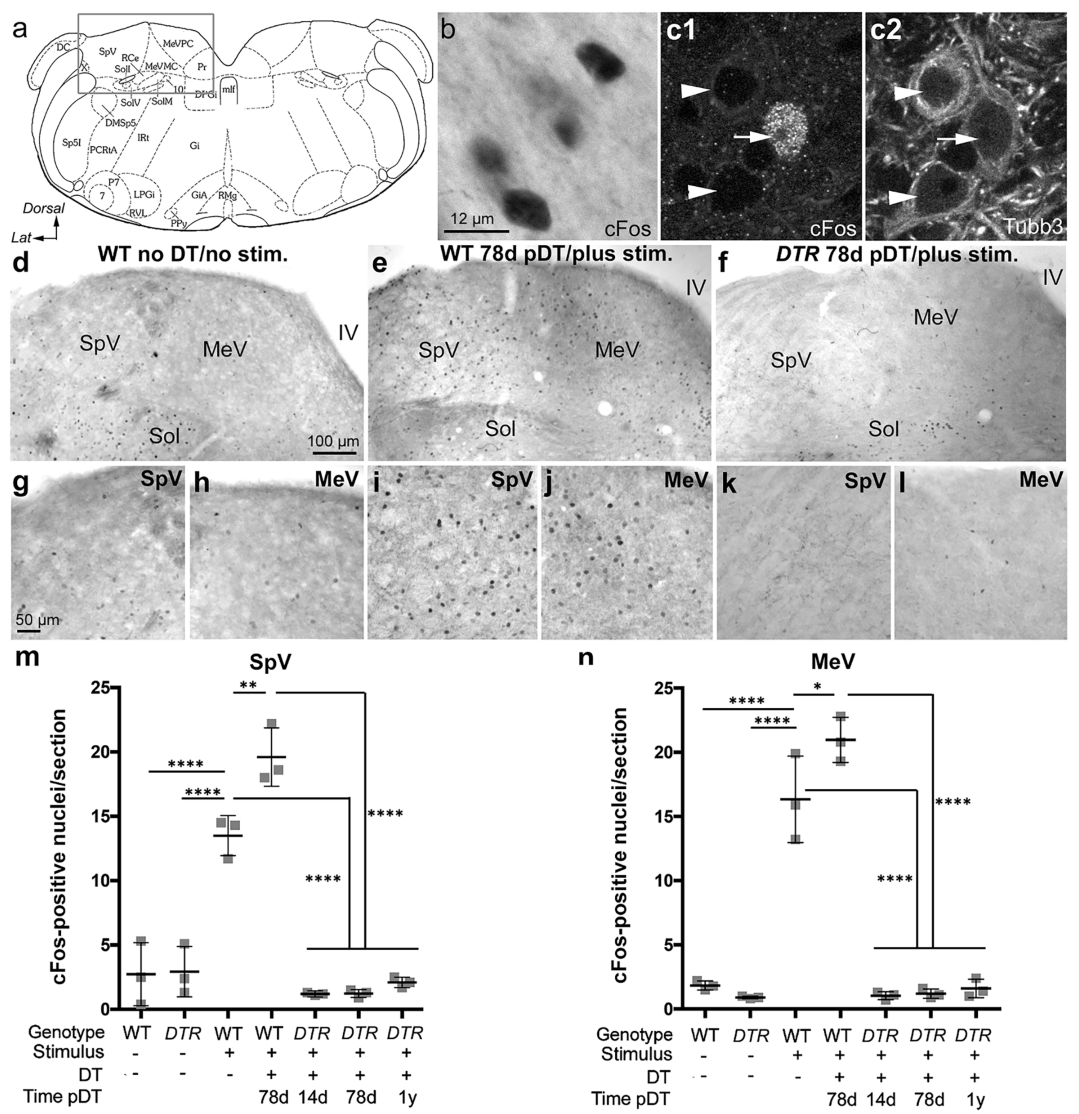
Sinusoidal centrifugation increased nuclear cFos VNNs in control, undamaged mice but not in *DTR* mice after 50 ng/g DT. **(A)** The boxed area in this schematic adopted from Paxinos and Franklin (29) shows the approximate region in the brainstem for images in b-l. **(B)** Brightfield image of cFos labeling (black) in nuclei of neurons in the MeV nucleus from a WT mouse with no DT treatment. **(C1)** High magnification image showing a cFos-labeled nucleus (white) in a neuron (arrow). **(C2)** Shows Tubb3 labeling (white) in the same field as c1. Arrowheads in c1, c2 point to two cFos-negative neurons. **(D,G,H)** A section from a WT mouse that received no DT and no centrifugation (stim) shows few cFos-labeled neurons in SpV **(G)** and MeV **(H)** nuclei. **(E,I,J)** A section from a WT mouse that underwent centrifugation at 78d post (p)-DT had a significant number of cFos labeled neurons in SpV **(I)** and MeV **(J)** nuclei. **(F,K,L)** A section from a *DTR* mouse that underwent centrifugation at 78d post-DT shows very few cFos-labeled cells in SpV **(K)** and MeV **(L)** nuclei. In all three mice **(D–F)**, the solitatius tract (sol), served as an internal positive control for cFos labeling. **(M,N)** Graphs show mean (±SEM) numbers of cFos-labeled neurons per section for each nucleus. Each gray square is the mean from a single mouse. Data for the two nuclei were obtained from the same mouse. One-factor ANOVA revealed significant differences, with lines indicating each comparison, and asterisks showing the significance level for each comparison (*p ≤ 0.05, **p ≤ 0.01, ****p ≤ 0.0001). 7, facial nucleus; 10, vagus nucleus; DC, dorsal cochlear nucleus; MeVMC, medial vestibular nucleus, magnocellular division; DMSp5, dorsomedial spinal trigeminal nucleus; DPGi, dorsal paragigantocellular nucleus; IRt, intermediate reticular nucleus; Gi, nucleus reticularis gigantocellularis; GiA, gigantocellular reticular nucleus alpha; LPGi, lateral paragigantocellular nucleus; MeVPC, medial vestibular nucleus, parvocellular division; P7, perifacial zone; Pr, prepositus nucleus; Ppy, parapyramidal nucleus; mlf, medial longitudinal fasciculus; PCRtA, parvicellular reticular nucleus, alpha; Rce, raphe cap; RMg, raphe magnus; RMg, raphe magnus nucleus; RVL, rostroventrolateral reticular nucleus; Sol, solitary nucleus (ventrolateral VL, ventral V, IM intermediate, and medial M subdivisions); Sp5I, spinal trigeminal nucleus, pars interpolaris; SpV, spinal vestibular nucleus; X, nucleus x.

**Figure 5:**
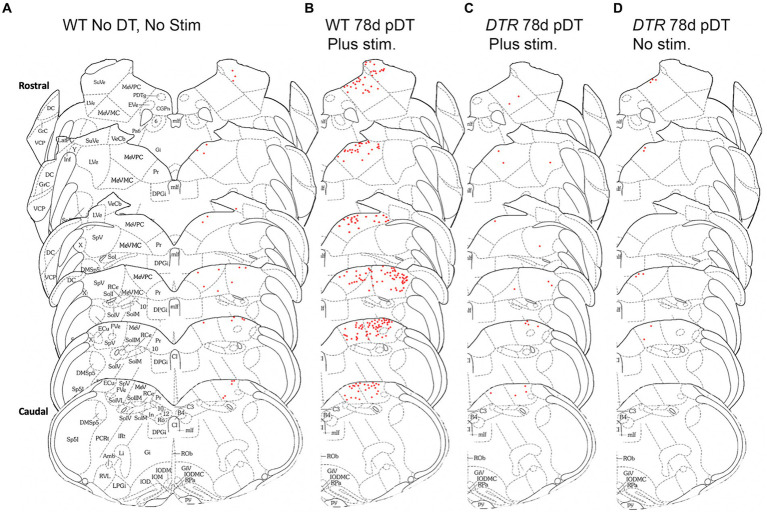
After sinusoidal centrifugation, numbers of cFos-labeled VNNs increased in MeV and SpV nuclei. The distribution of cFos labeled neurons (red dots) in VNNs is shown for one representative mouse from each group using schematics derived from Paxinos and Franklin (29). **(A)** WT mouse that received neither DT nor centrigugation (stim). **(B)** WT mouse that underwent centrifugation at 78d post-DT. **(C)** *DTR* mouse that experienced centrifugation at 78d post-DT. **(D)** *DTR* mouse at 78d post-DT that did not undergo centrifugation. Abbreviations for the schematics are defined in Figure 4.

**Figure 6:**
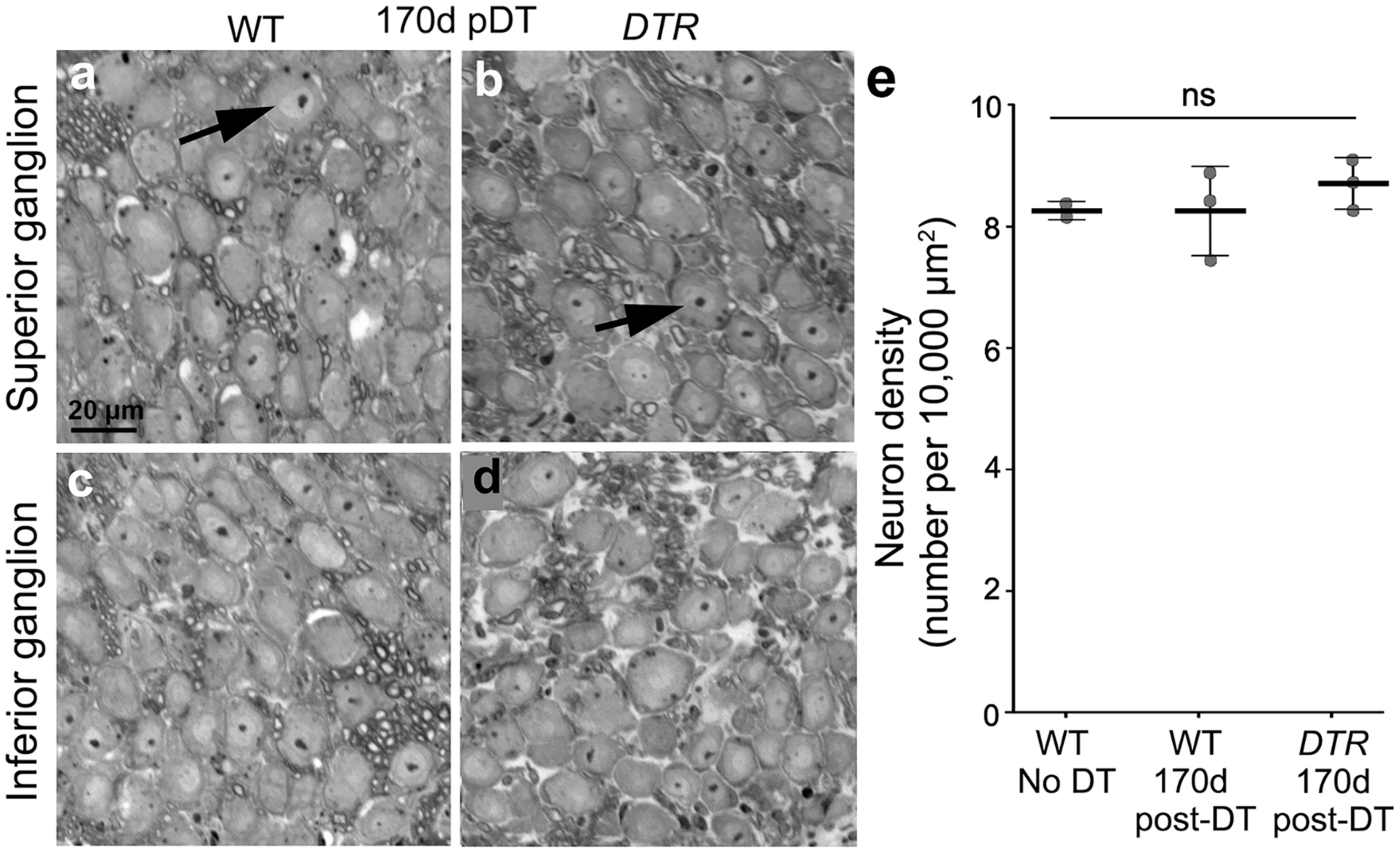
VGNs persisted after treatment with 50 ng/g DT. **(A–D)** Representative cross-sections through the vestibular ganglion from WT mouse **(A,C)** and a *DTR* mouse **(B,D)**, both at 170d post-DT. Neurons from the superior segment of the vestibular ganglion are shown in panels **(A,B)**, and neurons from the inferior segment are shown in panels **(C,D)**. **(E)** Graph shows mean (±SEM) neuron density (neurons per 10,000 μm^2^) for WT mice with no DT, WT mice at 170d post-DT, and *DTR* mice at 170d post-DT. Each gray circle is the mean from a single mouse. One-way ANOVA showed no significant differences (ns) between groups.

**Figure 7:**
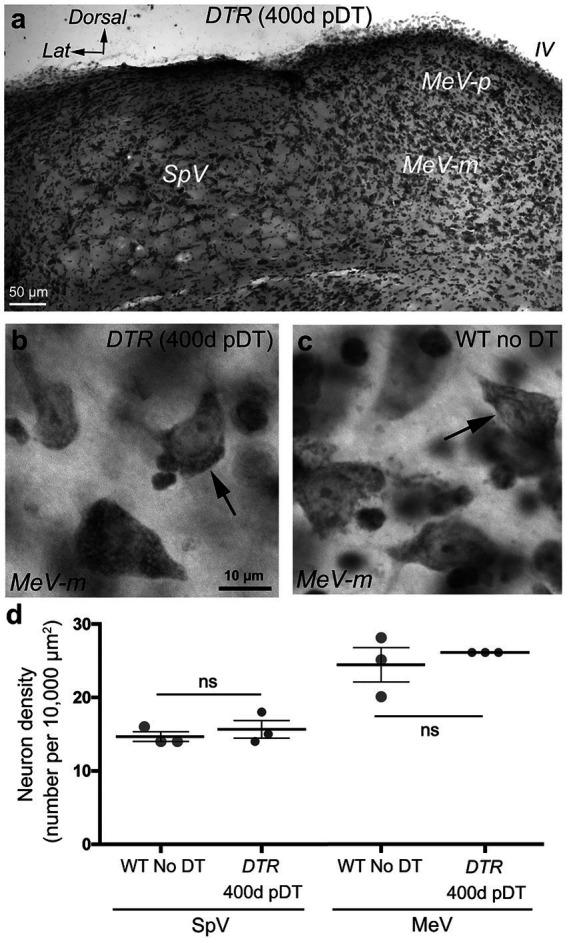
VNNs persisted after treatment with 50 ng/g DT. **(A)** Nissl-stained coronal section through SpV and MeV nuclei of a *DTR* mouse at 400d post- DT. **(B)** High magnification image of the magnocellular region of MeV nucleus (MeV-m) of a *DTR* mouse at 400d post-DT. Arrow points to a neuron. **(C)** High magnification image of the MeV-m nucleus of a WT mouse not treated with DT. Arrow points to a neuron. **(D)** Graph shows mean (±SEM) neuron density (number of cells per 10,000 μm^2^) in SpV and MeV nuclei of WT mice not treated with DT compared to *DTR* mice at 400d post-DT. Each gray circle is the mean from a single mouse. *T*-tests showed no significant differences (*ns*) between the two groups for each nucleus. IV, 4th ventricle.

**Figure 8:**
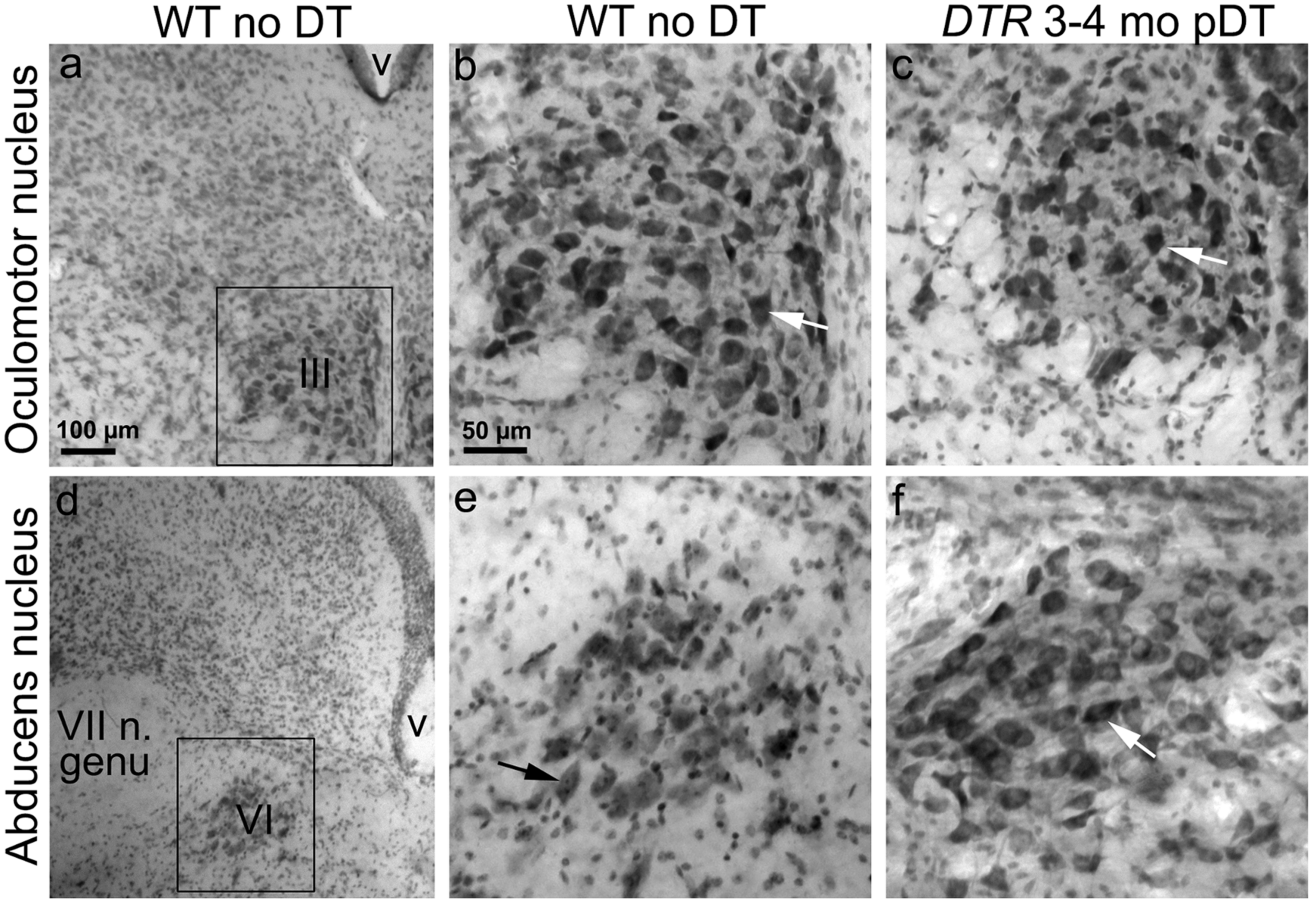
Oculomotor nuclei showed no loss of neurons after 50 ng/g DT. Nissl-stained coronal sections through oculomotor nucleus (III, **A–C**) and abducens nucleus (VI, **D–F**). **(A,D)** show low magnification views of each nucleus in a WT mouse with no DT. **(B,E)** show high magnification views of the boxed regions in panels **(A,D)**. **(C,F)** show high magnification views of each nucleus from a *DTR* mouse at 400d post (p)-DT in regions similar to **(B,E)**. VII n. genu = internal genu of facial nerve (near abducens nucleus). V, ventricle.

**Figure 9:**
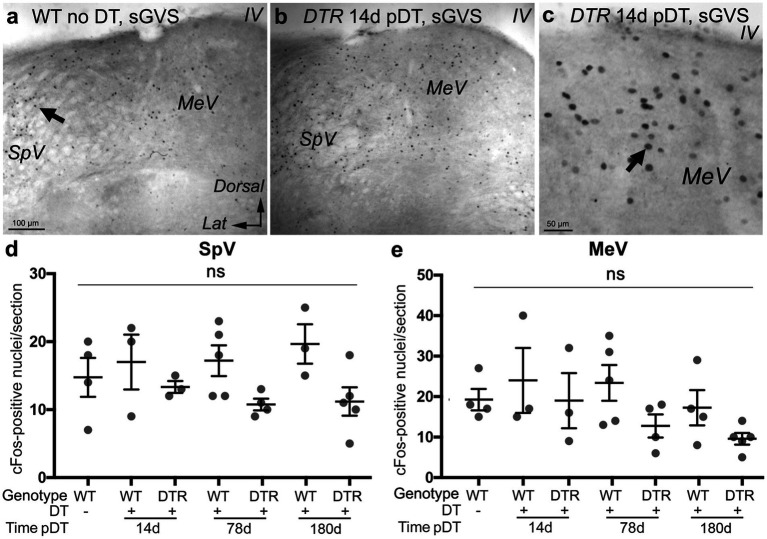
sGVS evoked cFos in VNNs even after near-complete HC destruction using 50 ng/g DT. **(A)** A representative low-magnification coronal section showing cFos immunolabeling in SpV and MeV nuclei after sGVS in a WT mouse that received no DT. **(B)** cFos labeling in a similar region from a *DTR* mouse at 14d post (p)-DT. **(C)** Higher magnification of a similar region of MeV nucleus as shown in panel **(B)**. **(D,E)** Graphs show the mean (±SEM) number of cFos-labeled nuclei per section in the SpV **(D)** and MeV **(E)** nuclei in WT mice after sGVS with no DT treatment, WT mice after sGVS at 14d, 78d, or 1 year post-DT, and in *DTR* mice after sGVS at 14d, 78d, or 1 year post-DT. Each gray circle is the mean from a single mouse. One-way ANOVA showed no significant differences (ns) between groups. Lat, lateral. IV, 4th ventricle.

In all control groups, utricles (Figures 1A,F) and horizontal ampullae (Figures 2A,F) had around 3,600 and 850 HCs (type I plus II) per organ, respectively (Table 1). Normal HC counts were measured in WT mice as late as 140d post-DT and in *DTR* mice that did not receive DT. In *DTR* mice at 14d post-DT, there was significant HC loss throughout utricles (Figure 1B) and horizontal ampullae (Figure 2B) to 9 and 6% of controls, respectively (Figures 1F, 2F; Table 1). These mice exhibited abnormal vestibulo-motor behaviors including head-bobbing, unsteady gait, circling, and spinning when lifted by their tails (data not shown), as described before (11). In utricles, one-way ANOVA showed that HC numbers increased significantly by 70d post-DT relative to 14d (compare Figures 1B,D,F; Table 1). HC numbers did not change significantly between 70d and 395d post-DT (Figure 1F; Table 1). In horizontal ampullae, HC numbers increased significantly between 42d and 170d post-DT (compare Figures 2C,E,F; Table 1). At 170d pDT, HC numbers were 21 and 14% of controls in utricles and horizontal ampullae, respectively. HC addition appeared to be most concentrated in peripheral zones of both types of organs (Figures 1B–E, 2B–E). Vestibulo-motor deficits were still evident at 170d pDT. These findings are consistent with those reported previously in mice with a similar genetic background (11, 34, 35).

**Table 1:** High-dose DT (50 ng/g): Counts for all HCs (type I plus type II).

UTRICLE (data for Figure 1F)					
Genotype	Time	HCs per organ (Avg ± SD)	% of control*	N	SIG. DIFFS. PER ANOVA
Wildtype	No DT	3,624 ± 167	–	4	*p* ≤ 0.0001 for DTR 14, 42, 70, 140, 170, 395d
	14d pDT	3,607 ± 215	–	3	" " " " " " "
	140d pDT	3,609 ± 148	–	3	" " " " " " "
*DTR*	No DT	3,646 ± 275	–	4	" " " " " " "
	14d pDT	310 ± 58	9%	7	*p* ≤ 0.0001 for all controls & DTR 70, 140, 170d
	42d pDT	518 ± 36	14%	5	*p* ≤ 0.0001 for all controls
	70d pDT	713 ± 188	20%	11	*p* ≤ 0.0001 all controls & DTR 14d
	140d pDT	727 ± 76	20%	8	*p* ≤ 0.0001 all controls & DTR 14d
	170d pDT	768 ± 58	21%	6	*p* ≤ 0.0001 all controls & DTR 14d
	395d pDT	580 ± 21	16%	3	*p* ≤ 0.0001 all controls

AMPULLA (data for Figure 2F)					
Genotype	Time	HCs per organ (Avg ± SD)	% of control*	N	SIG. DIFFS. PER ANOVA
Wildtype	No DT	890 ± 65	–	4	*p* ≤ 0.0001 for DTR 14, 42, 70, 140, 170d
	14d pDT	845 ± 19	–	3	" " " " " " "
	170d pDT	853 ± 12	–	3	" " " " " " "
*DTR*	No DT	887 ± 70	–	3	" " " " " " "
	14d pDT	55 ± 12	6%	6	*p* ≤ 0.0001 all controls
	42d pDT	45 ± 23	5%	4	*p* ≤ 0.0001 all controls; *p* ≤ 0.05 DTR 170d
	70d pDT	102 ± 26	12%	8	*p* ≤ 0.0001 all controls
	140d pDT	92 ± 9	11%	4	*p* ≤ 0.0001 all controls
	170d pDT	119 ± 22	14%	6	*p* ≤ 0.0001 all controls; *p* ≤ 0.05 DTR 42d

*WT, 14d pDT.

**Table 2:** High-dose DT (50 ng/g): Density of type I or type II HCs.

UTRICLE (Data for Figure 1G)								
Genotype	Time	Type I HCs*	SIG. DIFFS. PER ONE-WAY ANOVA: HCI***	Type II HCs*	SIG. DIFFS. PER ONE-WAY ANOVA: HCII***	N	HCI: % of control**	HCII: % of control**
Wildtype (WT)**	70d pDT	93 ± 17	*p* ≤ 0.0001 for DTR 14, 42, 70, 140, 395d	92 ± 21	*p* ≤ 0.01 for DTR 140d; *p* ≤ 0.001 for DTR 70; *p* ≤ 0.0001 for DTR 14, 42, 395d	3	–	–
*DTR*	14d pDT	11 ± 9	*p* ≤ 0.0001 for WT	10 ± 5	*p* ≤ 0.0001 for WT; *p* ≤ 0.05 for DTR 70d; *p* ≤ 0.01 DTR for 140d	3	12	11
	42d pDT	12 ± 5	*p* ≤ 0.0001 for WT	28 ± 10	*p* ≤ 0.0001 for WT	3	13	30
	70d pDT	6 ± 4	*p* ≤ 0.0001 for WT	41 ± 2	*p* ≤ 0.001 for WT; *p* ≤ 0.05 DTR 14d	3	6	45
	140d pDT	3 ± 2	*p* ≤ 0.0001 for WT	47 ± 5	*p* ≤ 0.01 for WT and for DTR 14d	3	3	51
	395d pDT	2 ± 1	*p* ≤ 0.0001 for WT	30 ± 1	p ≤ 0.0001 WT	3	2	33
AMPULLA (Data for Figure 2G)								
Genotype	Time	Type I HCs*	SIG. DIFFS. PER ONE-WAY ANOVA: HCI***	Type II HCs*	SIG. DIFFS. PER ONE-WAY ANOVA: HCII***	N	HCI: % of control**	HCII: % of control**
Wildtype (WT)**	14d (*n* = 1) & 42d pDT (*n* = 2)*	82 ± 9	*p* ≤ 0.0001 for DTR 14, 42d; *p* ≤ 0.001 for 170d	86 + 11	*p* ≤ 0.001 for DTR 170; *p* ≤ 0.0001 for DTR 14, 42d	3	–	–
*DTR*	14d pDT	15 ± 5	*p* ≤ 0.0001 for WT	6 ± 2	*p* ≤ 0.0001 for WT; *p* ≤ 0.01 for 42, 170d	3	19	8
	42d pDT	12 ± 4	*p* ≤ 0.0001 for WT	6 ± 3	*p* ≤ 0.0001 for WT	3	15	7
	170d pDT	19 ± 7	*p* ≤ 0.001 for WT	17 ± 4	*p* ≤ 0.001 for WT	2	9	41

*HCs per 10,000 μm^2^ (Avg ± SD).

***Shows groups significantly different from the group in gray shading on left.

**WT controls received 25 ng/g DT.

Next, we determined which type of HCs (I versus II) were destroyed and regenerated in *DTR* mouse utricles after high-dose DT using cell counting methods similar to Pujol et al. (31) and Bucks et al. (30). We employed myosin VIIa immunolabeling to assess the morphology of the HC’s hair bundle (stereocilia) and soma, and we used neurofilament (NF) immunolabeling to determine the type of VGN terminal(s) – calyx or bouton – on each HC. DAPI labeling was used to assess the shape and size of cell nuclei. Features of normal type I and II HCs are shown in confocal micrographs from control mice in Figures 1H–L, which indicates levels for each optical xy slice (parallel to the lumen) shown in panels H-K. Type I HCs were distinguished by long stereocilia (Figure 1H), a thin apical cytoplasm or ‘neck’ (Figure 1I), a small round nucleus (Figure 1J), and a nuclear position deep to type II HC nuclei (Figure 1J). Additionally, type I HC identify was confirmed by a calyx-type VGN terminal surrounding each type I HC (Figure 1K). Type II HCs had larger oval-shaped nuclei in the apical-most layer (Figure 1I) and cytoplasmic processes that emanate from their basolateral surfaces (Figure 1J). In Figure 1H, the short stereocilia of type II HCs are obscured by the long type I bundles. Note in Figure 1K that type II HCs lack a NF-labeled calyx afferent. In *DTR* mice after high-dose DT, most HCs at 70d post-DT and at later times had very short stereocilia (Figures 1M,P), oval nuclei (Figure 1N), and basolateral processes (Figures 1N,P), which are all characteristics of type II HCs. Only rare HCs at later timepoints were surrounded by a calyx afferent terminal (Figure 1O).

HC counts and one-way ANOVA (using data for either type I HCs or type II HCs) demonstrated that the density of type II HCs in *DTR* mice increased significantly between 14d and 70d post-DT compared to WT controls (Figure 1G; Table 2). Densities of type II HCs at 70d and 140d were 45 and 51% of controls, respectively. Type II HC density decreased to 33% of controls at 395d post-DT. This decrease was associated with a drop in mean total HC density at this later time (from 768 at 170d post-DT to 580 at 395 days post-DT; Table 1), but neither drop was statistically significant. In contrast, type I HC density in *DTR* mice did not change significantly after 14d post-DT to as late as 395d and were 3 and 2% of controls at 140d and 395d post-DT, respectively (Figure 1G; Table 2). Thus, at later recovery times after high-dose DT, utricles were populated mostly by regenerated type II HCs. Two-way ANOVA (time/genotype group x HC type) showed that HC type was a significant factor in variation amongst HC counts (*p* ≤ 0.0001). These findings resemble those reported previously in *DTR* mice (11, 34, 36), although this is the first time that type I and type II HC numbers have been rigorously quantified at recovery times greater than 12 weeks (84 days) using this method of HC destruction.

We also counted type I and type II HCs in horizontal ampullae after high-dose DT using the same criteria as for utricles (Figure 2G; Table 2). Sensory epithelium from an undamaged ampulla (WT, 14d post-DT) is shown in Figures 2H,I. Both type I and type II HCs were present and had normal appearances. Figures 2J,K show an epithelium after recovery from HC destruction (*DTR* mouse, 170d post-DT). Most HCs were type II-like, with short stereocilia and no calyx terminal. We detected only rare type I-like HCs, with long stereocilia and a clear calyx terminal. HC counts and one-way ANOVA confirmed that, while both type I and II HCs in horizontal ampulla were largely destroyed by high-dose, 50 ng/g DT, only type II HCs returned over time (Figure 2G; Table 2).

These observations are supported by Hicks et al. (34), who determined that, while many supporting cells in utricle, saccule, or horizontal ampulla transdifferentiated into type II HCs after high-dose DT in adult *DTR* mice, no supporting cells did so, even after 12 weeks (84d) post-DT.

### 3.2 The aVOR_H_ was lost after HC destruction caused by high-dose DT and did not return after type II HC regeneration

To begin to test if natural regeneration of type II HCs restores vestibular function after high-dose DT, we examined the angular vestibulo-ocular reflex (aVOR_H_), which relies on HCs in the horizontal ampulla. We measured eye velocity as mice underwent *en bloc* constant amplitude (±10°) sinusoidal rotation from 0.3 to 1.0 Hz about a vertical axis. Eye position was measured using video-oculography (VOG), and VOR gains (eye velocity/head velocity) were calculated.

In three sets of control, undamaged mice (many included in Figures 1, 2), average VOR gains ranged from 0.20 to 0.45 across frequencies (Figure 3). In contrast, *DTR* mice at 14d, which had near-complete HC destruction in the horizontal ampulla (Figure 2F), did not have measurable VOR except at 1.0 Hz. At 70d post-DT, *DTR* mice showed little or no VOR at 0.3 or 0.5 Hz and only small gains at 0.7 and 1.0 Hz. At 140-170d, when 41% of type II HCs had been regenerated and few type I HCs were present (Figure 1G), there were only negligible VOR gains at 0.5, 0.7, and 1.0 Hz. Thus, aVOR_H_ gains showed little or no recovery coincident with regeneration of 41% of type II HCs (and no type I HCs) after near-complete HC destruction.

### 3.3 Sinusoidal centrifugation failed to evoke cFos in VNNs after HC destruction caused by high-dose DT, and the cFos response did not return after type II HC regeneration

Upon learning that the aVOR_H_ was not restored after regeneration of half of the type II HCs, we wondered if VNNs could even respond to head motions under those conditions. We subjected freely moving mice to sinusoidal centrifugation and determined the extent to which VNNs in control and damaged mice upregulated nuclear cFos protein. As described in Materials and Methods (Section 2.4), this stimulus probably altered HC activity in multiple vestibular organs to some degree and triggered sustained vestibular afferent responses in a stochastic manner. Prior studies used cFos to assess changes in VNN activity after vestibular stimulation, with pioneering studies examining cFos in VNNs after centripetal acceleration in rats (20, 21).

Each mouse was placed in a 500 mL beaker on a rotating platform, subjected to sinusoidal centrifugation for 10 min, and euthanized at 45 min after the end of the stimulus. We labeled brainstem sections with antibodies to phosphorylated cFos, as described by Holstein et al. (27). We examined labeling along the entire caudo-rostral extent of the VN complex. However, we only counted VNNs in MeV and SpV nuclei because most increased cFos labeling was seen there, similar to following linear or angular acceleration of rats (19–21).

Figure 4A shows the region of the dorsal brainstem where most images were taken. As noted before (27, 37), the nucleus solitarius (Sol; Figures 4A,D–F) had cFos-immunoreactive neurons under baseline conditions and served as an internal positive control for cFos labeling.

Initial tests of the assay were conducted in undamaged control mice. In WT or *DTR* mice that received no DT and were not subjected to the vestibular stimulus (sinusoidal centrifugation), only a few VNNs (~2 per section) were cFos-positive in SpV or MeV nuclei (Figures 4D,G,H,M,N; WT mouse given no DT). In contrast, undamaged mice that underwent centrifugation had numerous VNNs (~13–20 per section) with strong nuclear cFos labeling in both nuclei (Figures 4E,I,J,M,N show WT mouse at 78d pDT). Figures 4B–C2 show cFos labeling from a stimulated WT mouse given no DT, counter-stained for the neural marker ßIII tubulin (Tubb3). These images confirm that cFos-positive cells were neurons and show that only some VNNs showed evoked cFos after centrifugation. We detected a significantly larger cFos response in VNNs in both nuclei in WT mice at 78d pDT than in WT mice without DT (Figures 4M,N). This was a surprise because our quantitative analyses indicated that both groups of control mice have similar numbers of HCs in utricles and horizontal ampullae (Figures 1F, 2F).

In *DTR* mice at 14d post-DT, when near-complete HC loss had occurred in utricles and horizontal ampullae, centrifugation induced very few cFos-positive neurons (~1 per section) in SpV or MeV nuclei (Figures 4M,N). Similar observations were made at 78d and 360d (1 year) post-DT, after type II HC regeneration (Figures 4F,K–N). At all times post-DT, numbers of cFos-labeled VNNs in *DTR* mice after centrifugation were not significantly different from mice that had undergone no centrifugation.

We mapped cFos-labeled VNNs across the rostro-caudal extent of SpV and MeV nuclei for one representative mouse in each group (Figure 5). Note that, in undamaged mice after centrifugation (WT, 78d post-DT; Figure 5B), some superior vestibular nucleus neurons were labeled, and in the MeV nucleus, labeled cells were located primarily in the more dorsal, parvocellular region (MeVPC); we only occasionally detected labeled neurons in the more ventral, magnocellular region (MeVMC).

In summary, we found that centrifugation-evoked changes in nuclear cFos in VNNs in the MeV and SpV were robust in mice with a normal population of type I and type II HCs but were rare or absent immediately after HC destruction or at later times when type II HCs had been regenerated and type I HCs had been largely depleted.

### 3.4 Vestibular ganglion neurons and vestibular nucleus neurons persist after HC destruction and regeneration induced by high-dose DT

One potential explanation for the failure of both the aVOR_H_ and centrifugation-evoked cFos in VNNs to return after near-complete HC loss could be death of neurons in the vestibular pathway, which hypothetically may occur as a direct result of high-dose DT or secondary to vestibular HC loss. To assess this, we examined VGNs and VNNs as well as neurons in the abducens and oculomotor nuclei, which mediate the aVOR_H_. We also immunolabeled Pou4f3 throughout the brainstem and midbrain as another assessment of neuronal vulnerability to DT in *DTR* mice.

We examined neurons in the superior and inferior portions of the vestibular ganglion in plastic sections from two sets of control mice (WT mice without DT and WT mice at 170d post-DT) and in one set of damaged mice (*DTR* mice at 170d post-DT). In all three groups, VGNs appeared similar, with a large cell body and a nucleus containing a single centralized nucleolus (Figures 6A–D; slices from a WT mouse and a *DTR* mouse, both at 170d post-DT). Cell counts revealed no significant difference in VGN density amongst the three groups (Figure 6E).

We examined neurons in the SpV nucleus and the MeV nucleus (parvocellular and magnocellular regions combined) in vibratome sections from two sets of mice: control mice (WT mice with no DT) and damaged mice (*DTR* mice at 400d pDT). We counted VNNs in the SpV nucleus at two levels: the genu of the facial nerve and the external cuneate nucleus (which are rostral and caudal portions of the SpV nucleus, respectively). We counted VNNs in the MeV nucleus at these levels as well as at the inferior cerebellar peduncle (which is a rostral portion of the MeV nucleus). Figure 7A shows a representative section used for counting. We detected no difference in the appearance or the density of VNNs between control and damaged groups (Figures 7B–D). In this same series of sections, we checked the oculomotor and abducens nuclei and found no qualitative evidence for neuronal loss in either nucleus (Figure 8). Furthermore, we saw no Pou4f3-immunoreactive neurons in the vestibular ganglion, vestibular nucleus, or the two oculomotor nuclei (not shown).

In summary, we detected no significant loss of primary vestibular afferent neurons (VGNs), their brainstem targets (VNNs), or neurons in the two oculomotor nuclei required for the aVOR_H_ following complete HC destruction. These observations strongly suggest that degeneration of vestibular pathway neurons is not an underlying cause for the persistent loss of the aVOR_H_ or centrifugation-evoked cFos in VNNs.

### 3.5 Sinusoidal galvanic vestibular stimulation (sGVS) elicits cFos accumulation in VNNs after high-dose DT

To further explore why *DTR* mice treated with DT lacked both the aVOR_H_ and centrifugation-evoked cFos, we considered the possibility that, although VGNs and/or VNNs were preserved after HC destruction, they may have been rendered dysfunctional. To test the ability of VGNs to transmit action potentials to VNNs, and for VNNs to respond to those signals, we subjected mice to sGVS at the mastoid, using methods similar to Cohen et al. (28) and Holstein et al. (27). GVS is thought to inhibit or activate all vestibular nerve units, depending on the polarity, and likely has a smaller effect on vestibular HCs (38, 39). Because GVS in humans triggers ocular roll, not nystagmus (40, 41), and sGVS in rats activates vestibulo-sympathetic responses associated with otolith function (27, 28, 42, 43), it has been postulated that sGVS impacts otolith afferents most strongly (44). However, Curthoys and MacDougall (45) showed that sGVS in rodents can trigger weak canal afferent responses.

We examined sGVS-activated cFos labeling of VNNs in control, undamaged mice and in mice at different times after HC destruction with high-dose DT. sGVS was delivered via subcutaneous electrodes behind each ear under deep anesthesia. One hour later, mice were euthanized, and brainstem sections were prepared for cFos immunohistochemistry. We found that mice with complete HC destruction and regeneration had no significant difference in numbers of cFos-labeled VNNs than control mice with no HC loss. In control mice (WT no DT), sGVS triggered cFos labeling in VNNs in SpV and MeV nuclei (Figure 9A). As expected, numbers of cFos-positive nuclei were significantly higher in these mice than in control mice without sGVS (compare Figures 9D,E with Figures 4M,N, WT no DT, no stimulus). We saw no differences in numbers of cFos-labeled VNNs among any undamaged controls (including WT mice at 14, 78, or 180d post-DT) and mice that underwent near-complete HC loss and type II HC regeneration (*DTR* mice at 14d, 78d, and 180d post-DT) (Figures 9B–E). The observation that sGVS-evoked cFos was maintained at 14d post-DT, when very few vestibular HCs were present, demonstrates that sGVS can drive cFos expression in VNNs, independent of HCs. Furthermore, the vestibular nerve retained its ability to drive changes in cFos in VNNs for at least 180d days (6 months) after HC destruction, although we were not able to tease out the specific contribution of the regenerated HCs to this later response.

It is important to note that we did not see altered cFos labeling in other brainstem regions outside the vestibular and cochlear afferent pathways (e.g., within the ventral half of the brainstem, see Figure 9A), indicating that galvanic currents were targeted to the region of the mastoid and likely did not directly stimulate brainstem neurons.

### 3.6 Mice with type I HC preservation following low-dose DT had VOR and cFos responses

So far, we demonstrated that regeneration of type II HCs after near-complete HC destruction does not reverse declines in function induced by near-complete HC destruction, including gross motor behaviors, the aVOR_H_, and vestibular stimulus-evoked changes in cFos in VNNs. We also showed that these deficits are likely not due to loss of VGN, VNN, or oculomotor nuclei neurons. These findings strongly suggest that the persistent vestibular functional deficits were due to inadequacies at the sensory organs.

**Figure 10:**
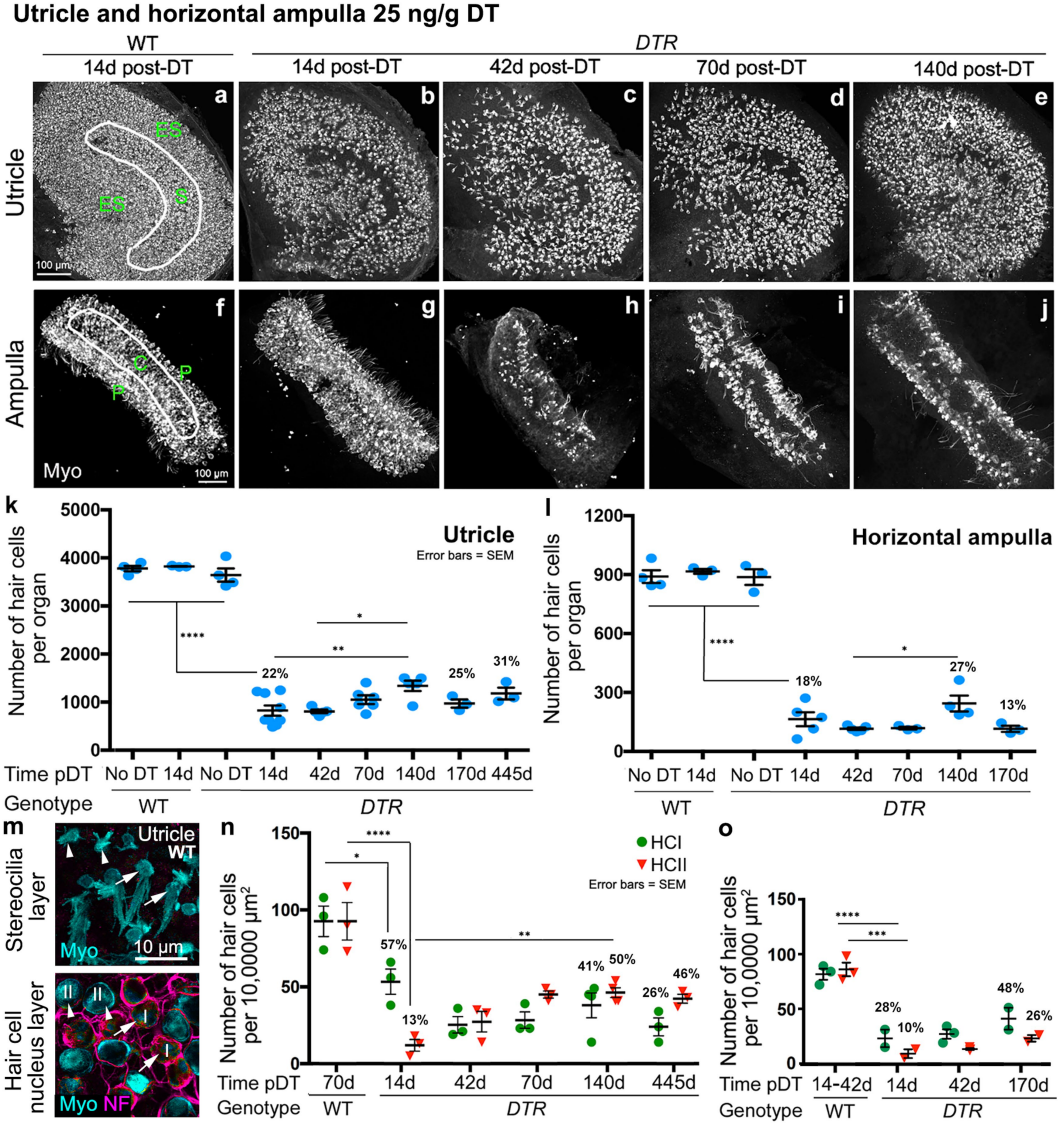
Low-dose, 25 ng/g DT causes similar type II HC destruction but spares about half of the type I HCs. **(A–J)** Images from whole utricles **(A–E)** and horizontal ampullae **(F–J)** labeled for the hair cell (HC) marker myosin VIIa (Myo, white) from WT mice at 14d post-DT **(A,F)** and from *DTR* mice at 14d **(B,G)**, 42d **(C,H)**, 70d **(D,I)**, and 140d **(E,J)** after 25 ng/g DT. Different utricle zones are indicated in panel **(A)**: ES, extrastriolar. S, striolar. C, central. P, peripheral. **(K,L)** Graphs show mean number (±SEM) of myosin VIIa-labeled HCs (both type I and II) for utricles **(K)** and horizontal ampullae **(L)** after 25 ng/g DT. Each graph shows data from three sets of control (undamaged) mice (WT with no DT, WT at 14d post-DT, and *DTR* mice with no DT) and for experimental (*DTR*, damaged) mice at times ranging from 14d to 445d for utricles and 14d to 170d post-DT for ampullae. **(M)** Confocal slices from one field in the extrastriolar utricle from a WT mouse at 42d after 25 ng/g DT taken in the xy plane (parallel to the lumen) at the levels of the hair bundles (top panel) and the HC nuclei (bottom panel). Utricle was labeled for myosin VIIa (Myo, cyan), neurofilament (NF, magenta) and DAPI (blue). Arrows point to two type I HCs, and arrowheads point to two type II HCs, at both levels. **(N)** Graph shows the mean (±SEM) number of utricular type I (green) and type II (red) HCs per 10,000 μm^2^ in WT mice at 70d post-DT and in *DTR* mice at 14d to 445d post-DT (all after 25 ng/g DT). **(O)** Graph shows the mean (±SEM) number of ampullary type I (green) and type II (red) HCs per 10,000 μm^2^ in WT mice at 14d-42d post-DT and in *DTR* mice at 14d to 170d post-DT (all after 25 ng/g DT). For all graphs, each symbol is the mean from a single mouse. % values represent % of control (also see Tables 3, 4). One-factor ANOVA revealed significant differences between some groups, with lines indicating comparisons and asterisks showing the significance level for each comparison (*p ≤ 0.05, **p ≤ 0.01, ***p ≤ 0.001, ****p ≤ 0.0001). To simplify the graphs, only the most pertinent significant differences are shown; additional significant differences are presented in Tables 3, 4. WT, wildtype; DTR, Pou4f3^DTR^; pDT, post-diphtheria toxin; Myo, myosin VIIa; I or HCI, type I hair cell; II or HCII, type II hair cell.

There are many potential causes of poor sensory organ function such as insufficient numbers of replacement HCs or insufficient synaptic function. Here, we opted to explore the paucity of type I HCs as a contributing factor. We tested whether preserving some type I HCs by using a low dose of DT would enable the VOR and stimulus-evoked cFos in VNNs. We gave *DTR* mice two injections of DT at 25 ng/g DT and examined utricles and horizontal ampullae using the same approach as for high-dose (50 ng/g) DT (see Figures 1, 2). Three undamaged control groups were used [WT mice with no DT, WT mice plus low-dose DT at 14d pDT (Figures 10A,F), and *DTR* mice with no DT]. HC numbers in controls are shown in Figures 10K,L and Table 3. In *DTR* mice, low-dose DT destroyed fewer HCs in both organs than high-dose DT (compare panels B-E,K and G-J,L in Figure 10; Table 3 with data in Figures 1B–E,F, 2B–E,F; Table 1).

**Table 3:** Low-dose DT (25 ng/g): Counts for all HCs (type I plus type II).

UTRICLE (Data for Figure 10K)					
Genotype	Time	HCs per organ (Avg ± SD)	% of control*	N	SIG. DIFFS.PER ANOVA
Wildtype	No DT	3,779 ± 114	–	4	*p* ≤ 0.0001 for DTR 14, 42, 70, 140, 170, 445d
	14d pDT	3,824 ± 13	–	3	" " " " " " "
*DTR*	No DT	3,646 ± 275	–	4	" " " " " " "
	14d pDT	826 ± 317	22%	5	*p* ≤ 0.0001 all controls and *p* ≤ 0.01 DTR 140d
	42d pDT	807 ± 78	21%	5	*p* ≤ 0.0001 all controls and *p* ≤ 0.05 DTR 140d
	70d pDT	1,051 ± 225	27%	3	*p* ≤ 0.0001 all controls
	140d pDT	1,339 ± 242	35%	4	*p* ≤ 0.0001 all controls; *p* ≤ 0.01 DTR 14d; *p* ≤ 0.05 DTR 42d
	170d pDT	971 ± 148	25%	3	*p* ≤ 0.0001 all controls
	445d pDT	1,180 ± 215	31%	3	*p* ≤ 0.0001 all controls

AMPULLA (Data for Figure 10L)					
Genotype	Time	HCs per organ (Avg ± SD)	% of control*	N	SIG. DIFFS. PER ANOVA
Wildtype	No DT	855 ± 37	–	2	*p* ≤ 0.0001 for DTR 14, 42, 70, 140, 170, 445d
	14d pDT	916 ± 21	–	4	" " " " " " "
*DTR*	No DT	887 ± 70	–	3	" " " " " " "
	14d pDT	164 ± 80	18%	5	*p* ≤ 0.0001 all controls
	42d pDT	115 ± 14	13%	5	*p* ≤ 0.0001 all controls and *p* ≤ 0.05 DTR 140d
	70d pDT	119 ± 11	13%	3	*p* ≤ 0.0001 all controls
	140d pDT	244 ± 81	27%	4	*p* ≤ 0.0001 all controls; *p* ≤ 0.05 DTR 42d
	170d pDT	115 ± 26	13%	3	*p* ≤ 0.0001 all controls

*WT, 14d pDT.

Analysis of utricular HC types in *DTR* mice at different times after low-dose DT revealed that type II HC density fell to 13% of undamaged controls and increased significantly to 50% of controls by 140d post-DT, similar to after high-dose DT, reflecting HC regeneration (compare Figures 10M,N; Table 4 with Figures 1G,M–O; Table 2). In contrast to high-dose DT, type I HC density was higher at all times. At 140d after low-dose DT, average densities of type I HCs were 41% of undamaged controls (Figure 10N; Table 4) compared to 2% after high-dose DT (Figure 1G; Table 2). Two-way ANOVA showed that HC type was not a significant factor in variation amongst HC densities (*p* > 0.05).

**Table 4:** Low-dose DT (25 ng/g): Density of type I or II HCs.

UTRICLE (Data for Figure 10N)								
Genotype	Time	Type I HCs*	SIG. DIFFS. PER ONE-WAY ANOVA: HCI***	Type II HCs*	SIG. DIFFS. PER ONE-WAY ANOVA: HCII***	N	HCI: % of control**	HCII: % of control**
Wildtype (WT)**	70d pDT	93 ± 17	*p* ≤ 0.05 for DTR 14d; *p* ≤ 0.01 DTR for 140d; *p* ≤ 0.001 DTR for 42, 70, 445d	92 ± 21	*p* ≤ 0.01 for DTR 70d; *p* ≤ 0.001 for DTR 140, 445d; *p* ≤ 0.0001 for DTR 14, 42d	3	–	–
*DTR*	14d pDT	53 ± 14	*p* ≤ 0.05 WT	12 ± 17	*p* ≤ 0.0001 WT; *p* ≤ 0.05 for DTR 70, 445d; *p* ≥ 0.01 for 140d	3	57	13
	42d pDT	25 ± 10	*p* ≤ 0.001 WT	27 ± 11	*p* ≤ 0.0001 WT	3	27	29
	70d pDT	28 ± 9	*p* ≤ 0.001 WT	45 ± 4	*p* ≤ 0.01 WT	3	30	49
	140d pDT	38 ± 16	*p* ≤ 0.01 WT	46 ± 6	*p* ≤ 0.001 WT; *p* ≤ 0.05 DTR 14d	4	41	50
	445d pDT	24 ± 10	*p* ≤ 0.001 WT	42 ± 5	*p* ≤ 0.001 WT	3	26	46
AMPULLA (Data for Figure 10O)								
Genotype	Time	Type I HCs*	SIG. DIFFS. PER ONE-WAY ANOVA: HCI***	Type II HCs*	SIG. DIFFS. PER ONE-WAY ANOVA: HCII***	N	HCI: % of control**	HCII: % of control**
Wildtype (WT)**	14d (*n* = 1) & 42d pDT (*n* = 2)	82 ± 9	*p* ≤ 0.01 for DTR 14, 42d; *p* ≤ 0.05 for DTR 170d	86 + 11	*p* ≤ 0.0001 for DTR 14, 42d; *p* ≤ 0.001 for DTR 170d	3	–	–
*DTR*	14d pDT	23 ± 11	*p* ≤ 0.01 for WT	9 + 6	*p* ≤ 0.0001 for WT	2	28	10
	42d pDT	27 ± 8	*p* ≤ 0.01 for WT	14 + 2	*p* ≤ 0.0001 for WT	3	33	16
	170d pDT	40 ± 13	*p* ≤ 0.05 for WT	22 + 5	*p* ≤ 0.001 for WT	2	48	26

* HCs per 10,000 μm^2^ (Avg ± SD). *** Shows groups significantly different from the group in gray. Shading on left. ** WT controls received 25 ng/g DT.

We made similar observations for HCs in horizontal ampullae after low-dose DT (Figure 10O): type II HC density fell and then increased over time (although not significantly) to 26% of controls, similar to after high-dose DT, most likely reflecting regeneration, and type I HC density was higher at all times compared to after high-dose DT (compare Figure 10O; Table 4 with Figure 2G; Table 2). At 170d post-DT, average densities of type I HCs were 48% of controls (Figure 10O; Table 4) compared to 9% after high-dose DT (Figure 2G; Table 2). Because there was no significant increase in type I HC density over time post-DT, and because no type I regeneration occurred after high-dose DT [e.g., Hicks et al. (34)], we suspect that type I HCs in both organs had survived the DT treatment.

*DTR* mice that received low-dose DT dose still exhibited stereotypical vestibulo-motor dysfunction behaviors such as head-bobbing, staggered gait, circling, and spinning when lifted via their tails, but these behaviors appeared less pronounced than *DTR* mice treated with high-dose DT.

**Figure 11:**
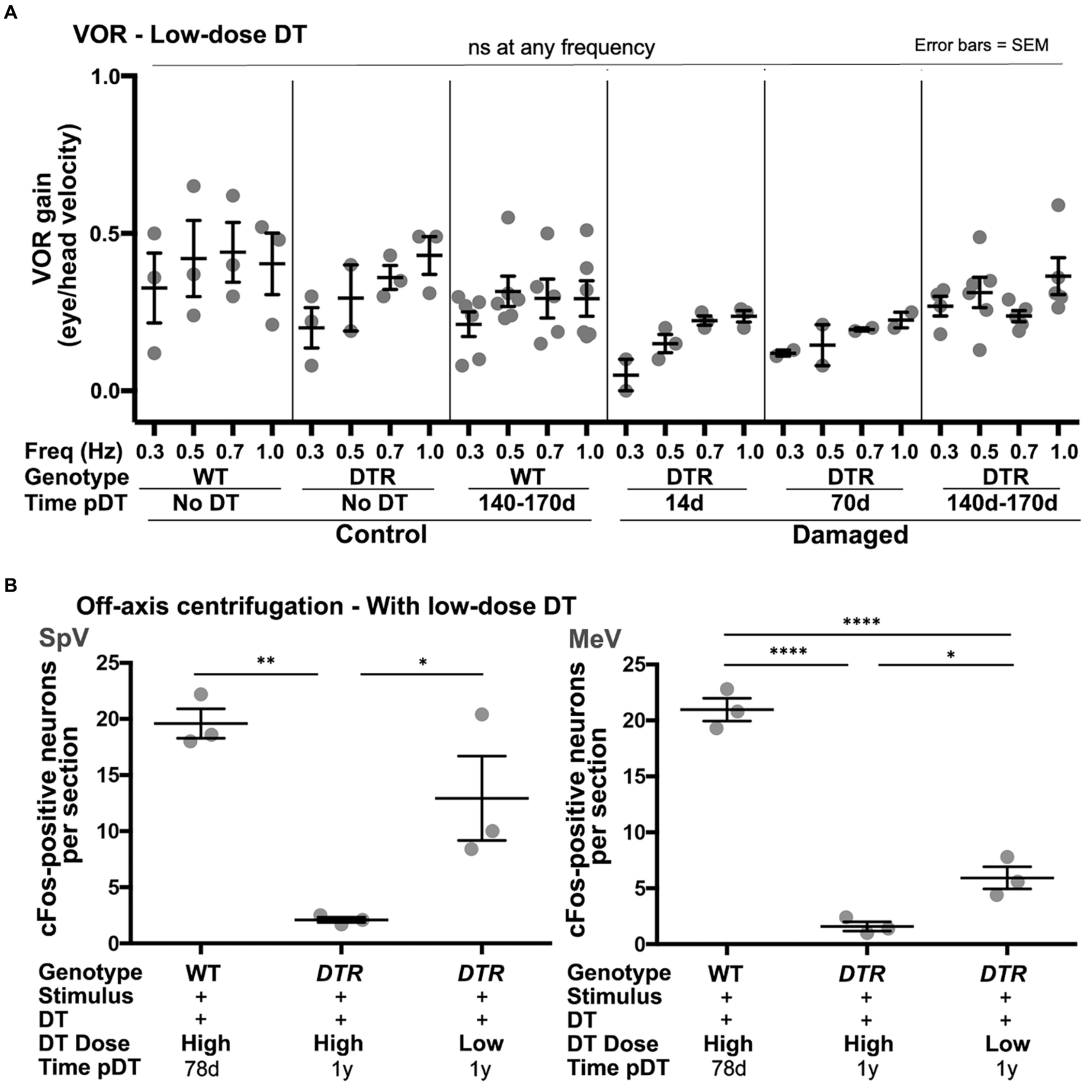
Following low-dose, 25 ng/g DT, VOR gains were preserved and centrifugation-evoked some cFos in VNNs. **(A)** Graph shows average (±SEM) aVOR_H_ gains following head rotations in the dark at a range of frequencies (Freq, 0.3 to 1.0 Hz) for three sets of control mice (WT mice with no DT, DTR mice with no DT, and WT mice at 140-170d post-DT) and three sets of experimental mice (*DTR* mice at 14, 70, and 140-170d post-DT). Each gray dot is the mean from a single mouse. One-factor ANOVA for each frequency revealed no significant differences (ns) between groups. **(B)** Graphs show average (±SEM) numbers of cFos-labeled neurons per section of each group; SpV nucleus data are shown on left and MeV nucleus data are shown on right. Each gray dot is the mean from a single mouse. One-factor ANOVA revealed significant differences, with lines indicating each comparison, and asterisks showing significance level for each comparison (*p ≤ 0.05, **p ≤ 0.01, ****p ≤ 0.0001).

We analyzed the aVOR_H_ in *DTR* mice that received low-dose DT and therefore had more surviving type I HCs. At each frequency, mean VOR gains at 14d and 70d post-DT were approximately half of undamaged controls (Figure 11A). At 140-170d post-DT, mean VOR gains at each frequency were closer to undamaged mice than at earlier times post-damage, suggesting some recovery of the VOR had occurred. Despite these trends, one-way ANOVA for data at each frequency showed no significant differences between any groups, presumably due to inter-animal variability. Regardless, these findings are in stark contrast to observations of *DTR* mice after high-dose DT in which VOR gains were close to zero at 14d post-DT and failed to improve as late as 140-170d post-DT (Figure 3).

Similar to the aVOR_H_, preserving approximately half of the type I HC population during type II HC regeneration resulted in larger VNN cFos responses to centrifugation compared to mice with primarily regenerated type II HCs present. *DTR* mice at 1 year after low-dose DT had significantly more centrifugation-evoked cFos in SpV and MeV VNNs than *DTR* mice at 1 year after high-dose DT (Figure 11B). Strikingly, in the SpV nucleus, there was no significant difference in centrifugation-evoked cFos responses between undamaged mice (WT at 78d post-DT) and *DTR* mice at 1 year after low-dose DT.

These observations demonstrate that preservation of approximately half of the type I HC population in utricles and horizontal ampullae is correlated with improved VOR gains and centrifugation-evoked cFos in VNNs, implying that type I HCs that survive damage make important contributions to these vestibular functions.

## 4 Discussion

### 4.1 Various assessments of the ascending vestibular pathway suggest it remains intact after near-complete vestibular HC destruction and subsequent type II HC regeneration

Our analysis of utricles and horizontal ampullae following HC destruction confirmed prior studies showing that hundreds of type II HCs are naturally regenerated in each organ and that few, if any, type I HCs are restored (11, 30, 34, 36). We found no significant loss of vestibular ganglion neurons (VGNs) or medial and spinal vestibular nucleus neurons (VNNs) several months after HC destruction. Most likely, synaptic activity or neurotrophic factors derived from the rapidly regenerated type II HCs and/or the rare DT-resistant type I HCs maintained VGNs, but it is also possible that trophic factors derived from supporting cells contributed to VGN survival. sGVS at the mastoid evoked cFos accumulation in VNNs up to 180 days after HC destruction. Therefore, the vestibular nerve can transmit action potentials that modulate VNN activity following HC destruction and type II HC regeneration. These observations bode well for studies that seek to restore vestibular function in adult mammals, such as by stimulating more complete forms of HC regeneration.

### 4.2 cFos labeling is evoked in VNNs during centrifugation of freely moving mice

To determine if head motions can induce VNN responses in adult mice after HC regeneration, we used cFos protein to test altered neural activity. cFos has been used in rats to assess VNN responses to sGVS (27) and in rat, cat, and monkey to examine VNN responses to body or head motions that stimulated vestibular otolithic and/or canal organs [for example, (17–21)]. We subjected unrestrained and undamaged adult mice to earth-vertical axis centrifugation for 10 min and found that this increased the number of cFos-labeled VNNs within 45 min. To our knowledge, this is the first use of cFos to measure vestibular stimulus-evoked VNN responses in mice in the context of targeted HC damage and regeneration. Increased cFos protein reflects altered transcriptional activity and therefore a change in a neuron’s state (46). We suspect that our centrifugation stimulus triggered changes in HC receptor potentials across multiple vestibular organs in a stochastic manner, altering vestibular nerve firing and thereby changing VNN activity. cFos protein accumulated in many but not all neurons in the SpV nucleus and in the dorsal-most part of the MeV nucleus (likely parvocellular neurons). A similar observation was made after a different type of centrifugation stimulus in rodents [for example (20, 21)]. We saw considerably less cFos activation in magnocellular MeV neurons or in lateral and superior vestibular nuclei. Partial cFos activation among VNNs in the VN complex was not surprising, given that cFos protein is not increased in all cells whose activity is altered by a given stimulus (15). We did not restrict head motions during centrifugation, so we cannot be sure which organs were stimulated or for how long. Regardless, there was relatively little variation in numbers and distribution of cFos-labeled VNNs in each group of mice, illustrating the reliability of this assay.

Surprisingly, we noted a significantly larger cFos response in VNNs in WT mice at 78d pDT than in WT mice without DT. The reason for this difference is not clear because HC numbers are similar in both groups. An increase in VNN cFos has been noted after unilateral labyrinthectomy in rodents [for example (47–49)]. But, we have no evidence from this study or a prior study (11) that vestibular HC activity is impacted in WT mice after DT treatment. It is notable that cochlear inner HCs in WT mice may be killed by DT treatment (23), raising the possibility that their loss somehow impacts VNN activity. It is also plausible that the two groups of mice had different patterns of evoked cFos because they behaved differently in the beaker during centrifugation.

We acknowledge that our assessment of cFos protein at 45 min after centrifugation does not inform on the full nature of VNN responses to this stimulus. For instance, there is poor temporal resolution of cFos expression using a protein assay. cFos protein levels can increase within 15 min of stimulation (50) but can be delayed by hours (51). An additional limitation of our study is we did not examine centrifugation-evoked cFos in cerebellar neurons that receive direct inputs from vestibular end organs (flocculonodular lobe or caudal vermis, primarily). Analysis of cFos in these regions will further inform on whether neurons in the ascending vestibular pathway can respond to vestibular stimuli when only regenerated type II HCs are present.

### 4.3 VOR and stimulus-evoked VNN cFos fail to recover with only regenerated type II HC present but are present when type I HCs are preserved

Although the vestibular sensory pathway remains largely intact after near-complete HC destruction, two measures of vestibular function – the aVOR_H_ and centrifugation-evoked cFos in VNNs – failed to improve after natural type II HC regeneration, even after 5 months (for VOR) or 1 year (for evoked cFos) of recovery. Our findings are consistent with other studies showing limited or no improvement in vestibular stimulus-evoked brainstem electrical potentials (VsEPs) after HC destruction using either gentamicin in guinea pigs (52) or 3,3′-iminodipropanenitrile in mice (53). Furthermore, Hirvonen et al. (54) saw no recovery of the VOR after HC destruction in chinchilla treated with gentamicin.

It is not clear why centrifugation fails to evoke cFos in VNNs after natural regeneration of type II HCs. One consideration is that otoconial function was lost upon HC destruction, as mice lacking otoconia do not have stimulus-evoked cFos in VNNs (55). Our observations speak against this possibility because *DTR* mice at several months post-DT have intact otoconia and underlying membranes (36). Furthermore, our finding that mice after low-dose DT could upregulate cFos upon centrifugation suggests that HCs maintain or re-establish their connection to the otoconial membrane after damage.

Our prior study (36) showed that regenerated HCs in utricles of adult *DTR* mice acquire many features typical of mature type II HCs, including polarized and property-oriented hair bundles and mechanoelectrical transduction (MET) currents. Furthermore, synaptic ribbon density and co-localization of synaptic ribbons with glutamate receptors in afferent boutons were within the normal range, suggesting the regenerated type II HCs may be capable of synaptic transmission (although this was not tested). However, on average, MET currents were smaller in regenerated HCs than native HCs, and regenerated HCs had smaller or absent HCN currents as well as persistent voltage-gated Na^+^ currents that were rarely detected in undamaged control mice. Thus, vestibular deficits may persist after severe damage because insufficient numbers of functionally mature (mechanoactive and synaptically active) type II HCs are replaced and head motions cannot evoke appropriate central responses in those conditions. The horizontal ampulla regenerates an even smaller percentage of type II HCs than utricles (14% versus 21%), which may help to explain why the aVOR_H_ does not recover.

Another feature of natural recovery in adult rodents is the failure to regenerate type I HCs (8–11, 34, 36). Type I and type II HCs differ in many respects including their molecular profiles, morphology, physiology, and innervation (56), yet their respective functions are not well defined. There are clues from birds that replacing type I HCs may be essential for restoring vestibular function. In chickens and pigeons, both type I and type II HCs are replaced after HC destruction (57–59). Over several months, regenerated HCs form new synapses upon VGNs (60), and some vestibular reflexes largely recover (61–64). Carey et al. (61) found that the magnitude of VOR gains after recovery in chickens was better correlated with numbers of regenerated type I HCs than regenerated type II HCs, which further supports the lack of type I HCs as a cause of lingering functional deficits in mammals after natural HC regeneration.

We explored whether type I HCs could enhance functional recovery in conjunction with type II HC regeneration in adult mice. Because we do not know how to drive type I HC regeneration, we used another approach: we gave adult *DTR* mice a lower DT dose, which killed the same number of type II HCs as the higher dose but spared half of the native type I HC population. This finding suggested that type II HCs are more sensitive to DT than type I HCs, which was surprising. After the low DT dose, the VOR was detected at 14d post-damage and persisted over time. This finding is in sharp contrast to the complete loss of the VOR after high-dose DT, when only regenerated type II HCs were present. In addition, mice that retained type I HCs had significantly more cFos-labeled neurons after centrifugation. Numbers of regenerated type II HCs were similar in the low- and high-dose groups, suggesting that improvements in vestibular responses were attributable to type I HC contributions, either by themselves or in combination with inputs from the regenerated type II HCs. However, there may be a significant difference between regenerated type II HCs in high-dose versus low-dose DT conditions that we are unaware of.

Altogether, our findings point to the importance of testing whether stimulating regeneration of at least half of the type I HC population, alone or in parallel with natural type II HC regeneration, would enhance recovery of vestibular function after HC loss in adult mammals. This knowledge should help investigators to develop better therapies for people with sensorineural vestibular deficits.

## Data availability statement

The original contributions presented in the study are included in the article/supplementary material, further inquiries can be directed to the corresponding author.

## Ethics statement

The animal study was approved by the Institutional Animal Care and Use Committee. The study was conducted in accordance with the local legislation and institutional requirements.

## Author contributions

EJ: Conceptualization, Data curation, Investigation, Methodology, Project administration, Writing – original draft, Writing – review & editing, Formal analysis, Validation. KS: Formal analysis, Methodology, Conceptualization, Data curation, Investigation, Project administration, Validation, Writing – original draft, Writing – review & editing. IB: Data curation, Investigation, Methodology, Project administration, Writing – original draft, Writing – review & editing, Formal analysis, Visualization, Validation. LP: Data curation, Formal analysis, Investigation, Methodology, Visualization, Writing – original draft, Writing – review & editing. HZ: Data curation, Formal analysis, Investigation, Methodology, Visualization, Writing – original draft, Writing – review & editing. CF: Data curation, Formal analysis, Investigation, Methodology, Visualization, Writing – review & editing. JoP: Data curation, Formal analysis, Writing – review & editing, Methodology, Validation. JG: Data curation, Formal analysis, Methodology, Validation, Writing – review & editing. TN: Data curation, Formal analysis, Methodology, Validation, Writing – review & editing, Supervision. JaP: Conceptualization, Data curation, Formal analysis, Funding acquisition, Investigation, Methodology, Project administration, Resources, Supervision, Validation, Writing – original draft, Writing – review & editing. JS: Data curation, Formal analysis, Methodology, Supervision, Validation, Writing – review & editing, Conceptualization, Funding acquisition, Investigation, Project administration, Resources, Visualization, Writing – original draft.
